# Chemistry and Bioactivity of the Deep-Water Antarctic Octocoral *Alcyonium* sp.

**DOI:** 10.3390/md20090576

**Published:** 2022-09-14

**Authors:** Anne-Claire D. Limon, Hiran M. L. W. Patabendige, Ala Azhari, Xingmin Sun, Dennis E. Kyle, Nerida G. Wilson, Bill J. Baker

**Affiliations:** 1 Department of Chemistry, University of South Florida, 4202 E. Fowler Avenue, CHE 205, Tampa, FL 33620, USA; 2 Department of Molecular Medicine, Morsani College of Medicine, University of South Florida, 12901 Bruce B. Downs Boulevard, MDC07, Tampa, FL 33612, USA; 3 USF Center for Global Health and Infectious Diseases Research, University of South Florida, 3010 USF Banyan Circle, IDRB 304, Tampa, FL 33612, USA; 4 Collections & Research, Western Australian Museum, 49 Kew Street, Welshpool 6106, Perth, WA 6106, Australia; 5 School of Biological Sciences, University of Western Australia, 35 Stirling Highway, Crawley, WA 6009, Australia

**Keywords:** alcyopterosin, *Clostridium difficile*, illudalane, *Leishmania donovani*, sesquiterpene

## Abstract

Chemical investigation of an Antarctic deep-water octocoral has led to the isolation of four new compounds, including three illudalane sesquiterpenoids (**1**–**3**) related to the alcyopterosins, a highly oxidized steroid, alcyosterone (**5**), and five known alcyopterosins (**4**, **6**–**9**). The structures were established by extensive 1D and 2D NMR analyses, while **9** was verified by XRD. Alcyopterosins are unusual for their nitrate ester functionalization and have been characterized with cytotoxicity related to their DNA binding properties. Alcyopterosins V (**3**) and E (**4**) demonstrated single-digit micromolar activity against *Clostridium difficile*, an intestinal bacterium capable of causing severe diarrhea that is increasingly associated with drug resistance. Alcyosterone (**5**) and several alcyopterosins were similarly potent against the protist *Leishmania donovani*, the causative agent of leishmaniasis, a disfiguring disease that can be fatal if not treated. While the alcyopterosin family of sesquiterpenes is known for mild cytotoxicity, the observed activity against *C. difficile* and *L. donovani* is selective for the infectious agents.

## 1. Introduction

Corals are encountered from the tropics to the polar seas, found on seamounts or geological formations up to 6000 m below the ocean’s surface [1,2]. In the south, corals are separated by the Antarctic Circumpolar Current from the contiguous oceans resulting in an ecological niche [3,4]. Biochemical knowledge of deep-water corals from Antarctica is impeded by the remoteness and extreme conditions required for access [5,6], leading to great interest in coral natural products for ecological and biomedical studies [7,8,9]. Past research suggests that deep-water coral species offer potential drug discovery resources from the terpenoids class, ranging from mono- to triterpenes [10,11,12]. Various cold-water terpenoids from deep-sea soft corals include the paesslerins [13], ainigmaptilones [14], and keikipukalides [6], many of which exhibit moderate cytotoxicity toward either human cancer cell lines or microbial pathogens [8].

**Figure 1:**
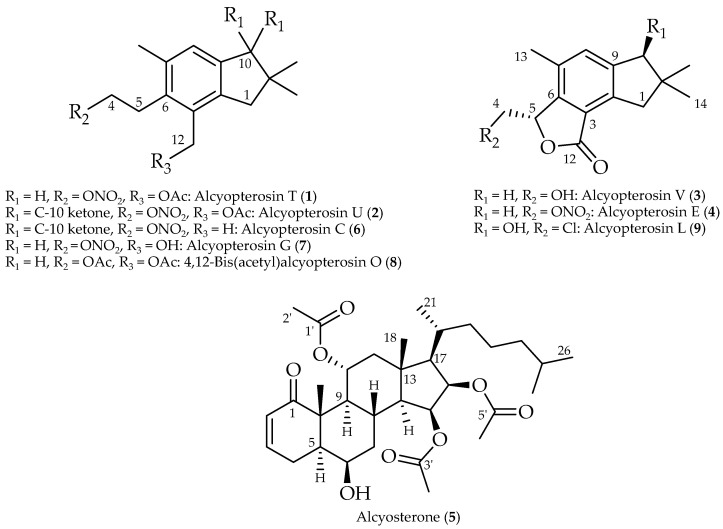
Terpenoids isolated from a deep-water Antarctic octocoral *Alcyonium* sp.

Originally found in fungi [15], illudalane sesquiterpenes have also been isolated from deep-sea corals [16,17] and marine sedimentary fungi [18]. Alcyopterosins are illudalane metabolites reported from the Antarctic soft corals *Alcyonium paessleri* and *A. grandis* that display terminal chlorine, hydroxyl, or nitrate ester moieties at the C-4 position of the aliphatic side chain [16,17]. Nitrate in seawater is considerably less abundant than, for example, the halides, so the appearance of a nitrate ester is unexpected and, to date, found exclusively in this class of marine natural products. We had the opportunity to study *Alcyonium* sp. from deep-water communities near Shag Rocks in the Scotia Arc of Antarctica. Six known alcyopterosins and three new ones (**1**–**3**) were obtained, in addition to a highly oxidized steroid, alcyosterone (**5**) (Figure 1). The metabolites were screened in a number of anti-infective assays and several showed promise against *Clostridium difficile* and *Leishmania donovani*.

## 2. Results and Discussion

Coral specimens were collected during a 2013 cruise to the Scotia Arc in the Southern Ocean near Shag Rocks, at a depth of between 126 and 130 m. Phylogenetic analysis was conducted on one specimen (WAM Z97931) using the *msh1* sequence. The coral clustered with other known *Alcyonium* spp. from the Southern Ocean region, but was divergent from those species (Figure S1), leading to its current identification as *Alcyonium* sp. indet.

The dichloromethane/methanol (1:1) extract of the freeze-dried coral was partitioned between ethyl acetate and water, and the lipophilic partition was separated using a gradient normal-phase medium pressure liquid chromatography (MPLC) system, yielding eight fractions. Several MPLC fractions were chosen for HPLC purification based on the characteristics of their ^1^H NMR spectra. In particular, the mid- and late-polar fractions displayed ^1^H NMR signals characteristic of the previously reported alcyopterosins [16,17], in particular the aromatic singlet (H-8) and a midfield oxymethylene (H_2_-4). Fractions F, G, and H, eluting roughly between 60–90% ethyl acetate in hexane, were found to harbor alcyopterosins E (**4**), C (**6**), G (**7**), 4,12-bis(acetyl)alcyopterosin O (**8**), and alcyopterosin L (**9**) (Table S1). Two new alcyopterosins (**1**, **2**) were found in the earlier eluting MPLC fractions, D and E, and fraction H was found to contain the previously undescribed hydrolysis product (**3**) of alcyopterosin E (**4**), along with **4**.

**Figure 2:**
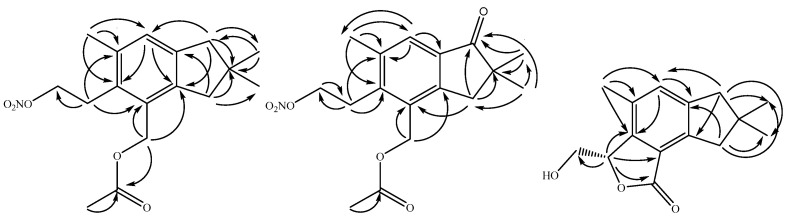
Key HMBC correlations establishing the planar structure of alcyopterosin T (**1**), alcyopterosin U (**2**), and alcyopterosin V (**3**).

Alcyopterosin T (**1**) displayed an HRESIMS [M + Na]^+^ at *m*/*z* 344.1460, which agrees well with C_17_H_23_NO_5_Na (calcd *m*/*z* 344.1468), and sharp IR bands at 1640 and 1280 cm^−1^ were consistent with the presence of a nitrate moiety. The ^1^H NMR spectrum (Table 1) displayed nine well-resolved signals, two of which were coupled triplets while the other seven were singlets. The HSQC spectrum identified the nine protonated carbon signals, and the additional seven non-protonated carbon signals were evident from the HMBC spectrum. Six carbon shifts in the olefinic region could be cyclized into an aromatic ring based on HMBC correlations (Figure 2) of the deshielded methyl group at δ_H_ 2.38 (C-13) to C-6 (δ_C_ 131.4), C-7 (δ_C_ 135.9) and C-8 (δ_C_ 128.0); H-8 (δ_H_ 7.06) to C-2 (δ_C_ 143.2), and C-6; H_2_-10 (*δ*_H_ 2.73) to C-2 and C-8; H_2_-1 (δ_H_ 2.79) to C-9 (δ_C_ 143.4); H_2_-12 (δ_H_ 5.16) to C-2 and C-3 (δ_C_ 131.1); and H_2_-5 (δ_H_ 3.15) to C-3 and C-7. Additional HMBC correlations between both H_2_-1 and H_2_-10 to C-11 (δ_C_ 40.4) and C-14/15 (δ_C_ 29.7) established a fused five-membered ring on the aromatic ring.

Two additional substitutions were found on the aromatic ring of alcyopterosin T (**1**). H_2_-12, besides the HMBC correlations described above in the aromatic ring, further correlated (Figure 2) to an ester-type carbonyl at δ_C_ 171.1 (C-1′), which could be elaborated into an acetate group based on the HMBC correlation of H_3_-2′ (δ_H_ 2.09) to C-1′. And lastly, H_2_-5 had both COSY correlations to H_2_-4 (δ_H_ 4.57) and HMBC correlation to C-4 (δ_C_ 63.2), completing the ^1^H and ^13^C assignments of **1**. Missing from the molecular formula is NO_3_, and the sole open valence on C-4 establishes alcyopterosin T as the acylated alcyopterosin G [16].

The spectral data for alcyopterosin U (**2**) were very similar to those of **1** and again reminiscent of the alcyopterosin family of metabolites. The HRESIMS ([M + H]^+^: *m*/*z* 336.1429; calcd for C_17_H_22_NO_6_: 336.1442) found that **2** has one additional oxygen and two protons fewer than **1**. The IR spectrum displayed the same sharp bands at 1640 and 1280 cm^−1^ supportive of the nitrate ester moiety, along with the absorptions at 1700 and 1750 cm^−1^ typical of ketone and ester functions, respectively [16]. The most obvious difference between the ^1^H NMR spectra of **1** and **2** was the absence of one methylene and the shift of the aromatic proton H-8, from δ_H_ 7.06 in **1** to 7.64 in **2**. The HMBC spectrum demonstrated a correlation between the *gem*-dimethyl protons (H_3_-14/15, δ_H_ 1.24) and a carbon signal at δ_C_ 211.4, reflecting a departure in **2** from the oxidation state of **1**. Taken with the missing methylene group in **2**, the ketone must be at C-1 or C-10. A methylene signal at δ_H_ 3.03 (H_2_-1) also correlated in the HMBC spectrum to the ketone, as well as δ_C_ 151.5 and 135.4. Because H_2_-12 (δ_H_ 5.25) also had an HMBC correlation to δ_C_ 151.5, but not to δ_C_ 135.4, then δ_C_ 151.5 must be C-2 and δ_C_ 135.4 must be C-9. An HMBC correlation between H_2_-1 and C-3 secured the position of the carbonyl at C-10. Further ^1^H and ^13^C shifts as well as HMBC correlations (Figure S10) supported the remaining substitution on the aromatic ring of **2** mirroring that observed for **1**.

The ^1^H NMR spectrum of alcyopterosin V (**3**) displayed a new pattern relative to those from **1** and **2**, though certain resemblances remained. Lacking an acetoxy signal found in **1** and **2**, the molecular formula of **3** was established as C_15_H_18_O_3_ from the HRESIMS, in conjunction with the ^13^C NMR spectrum (Table 1), (C_15_H_19_O_3_ [M + H]^+^: *m*/*z* 247.1328). The aromatic ring was established to be very much like that for **1**: from the HMBC, a significantly deshielded/aromatic proton at δ_H_ 7.24 (H-8) correlated with δ_C_ 141.1 (C-2 or C-9) and 142.4 (C-6), the latter of which also had HMBC correlation from highly deshielded/aromatic methyl at δ_H_ 2.37 (H_3_-13). The aromatic methyl showed further HMBC correlations to δ_C_ 130.0 (C-7) and 131.8 (C-8). With the observation of HMBC correlation of δ_H_ 5.55 (H-5) to C-6 and δ_C_ 122.5 (C-3), only C-2 and C-9 (δ_C_ 146.7 and 141.1) remained to secure as part of the aromatic ring. H-8, H_2_-1 (δ_H_ 3.04), and H_2_-10 (δ_H_ 2.74), the only hydrogen-bearing carbons near C-2 and C-9, are all 2 or 3 bonds apart and thus cannot assist in the assignment. Instead, we have assigned C-2 and C-9 based on their shift comparisons to similar carbons in **1** and **2**, but we note that they may be interchanged.

Substitution on the aromatic ring of **3** was completed by considering the HMBC correlations of the remaining protons and carbons. H_2_-1 and H_2_-10 were noted above as correlated in the HMBC with both C-2 and C-9, locating them on the ring relative to already established H_3_-13 and H-8; H_2_-10 was distinguished from H_2_-1 by HMBC correlation to C-8, disambiguating their relative positions. They also both correlated with C-11 (δ_C_ 40.9) and C14/15 (δ_C_ 28.8), completing the fused cyclopentane ring found on all the alcyopterosins. The final feature of alcyopterosin V was established by observation of the HMBC correlation of H-5 to both an oxymethylene (C-4, δ_C_ 63.2) and an ester-type carbonyl at δ_C_ 170.8 (C-12). As the protons of the oxymethylene (H-4a, δ_H_ 4.25; H-4b, δ_H_ 3.81) were COSY coupled to H-5, which was already affixed to the aromatic ring at C-6 as described above, the ester carbonyl must be located at C-3, completing a lactone ring. Insufficient material for optical spectra prevented comparison of the configuration of C-5 in **3** and alcyopterosin E (**4**), but **3** represents the nitrate ester hydrolysis product of **4**, due to which we suggest the two will share a common configuration. Additional support for the assigned configuration comes from an analysis of the coupling constants for the chiral proton H-5 of **3**, which match those of **4** in magnitude (**3**: ^3^*J*_4a-5_ = 2.5 Hz, ^3^*J*_4b-5_ = 6.1 Hz; **4**: ^3^*J*_4a-5_ = 2.3 Hz, ^3^*J*_4b-5_ = 6.6 Hz).

Further work was done to bring forward additional alcyopterosins, and a subsequent extraction was conducted and similarly fractionated. Alcyosterone (**5**) eluted late in the silica gradient (hexanes to ethyl acetate), suggesting a moderately polar metabolite. Upon analysis, it was determined to have the molecular formula C_33_H_50_O_8_ based on HRESIMS data that was corroborated by proton and carbon counts from their NMR spectra (Table 2). From the HRESIMS, the [M + H]^+^ was observed at *m*/*z* 575.3555, and [M − HOAc]^+^ was observed at *m*/*z* 515.3364. Analysis of the ^13^C NMR spectrum supported the 33 carbons accounted for by the MS and further indicated a ketone (C-1, δ_C_ 203.9), three ester-type carbons (C-1′, δ_C_ 169.4; C-3′, δ_C_ 170.4; C-5′, δ_C_ 169.9), two olefinic carbons (C-2, δ_C_ 128.4; C-3, δ_C_ 142.5), and four carbon signals in the oxygen-bearing region (C-6, δ_C_ 69.7; C-11, δ_C_ 70.4; C-15, δ_C_ 70.5; C-16, δ_C_ 73.0). The HSQC established the two olefinic carbons and all four of the oxygen-bearing carbons as methines and further indicated five aliphatic methines, six aliphatic methylenes, and eight methyl carbons. The ^1^H NMR spectrum provided few additional insights into this overview of alcyosterone other than to suggest that three of the methyl carbons were associated with acetate esters, based on their chemical shifts (H_3_-2′, δ_H_ 1.93; H_3_-4′, δ_H_ 2.06; H_3_-6′, δ_H_ 2.02) and HMBC correlation to their respective ester carbonyl.

**Figure 3:**
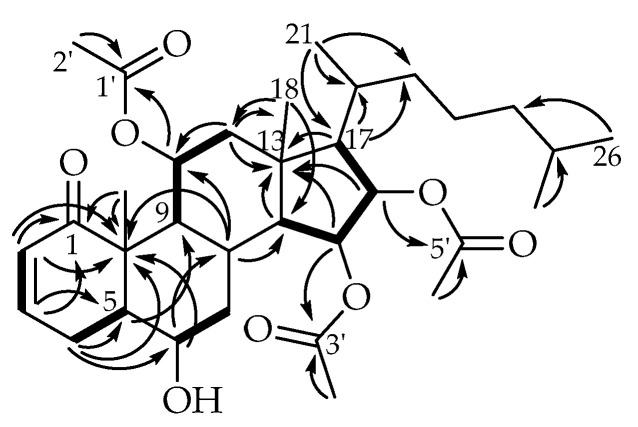
Key HMBC (→) and COSY (**—**) correlations for alcyosterone (**5**).

The chemical shift of H-3 (δ_H_ 6.58) and its associated carbon (C-3, δ_C_ 142.5) supported the presence of a conjugated system, which must be an α,β-unsaturated ketone. The HMBC strengthened that assignment as both H-2 (δ_H_ 5.83) and H-3 correlated with C-1 (δ_C_ 203.9) (Figure 3). H-3 was further correlated in the HMBC with methine C-5 (δ_C_ 46.6), while H-2 correlates to the quaternary C-10 (δ_C_ 47.7) and the methylene C-4 (δ_C_ 28.4). With correlations of H-4a (δ_H_ 2.79) and H-4b (δ_H_ 2.11) to C-2, C-3, C-5, and C-10, a six-membered ring was established bearing the aforementioned α,β-unsaturated ketone.

Extending the cyclohexenone, H_2_-4 further coupled in the HMBC spectrum to an oxymethine, C-6, and displayed a COSY correlation to H-5 (δ_H_ 1.86), the latter of which has an HMBC correlation with C-9 (δ_C_ 47.8). H-6 (δ_H_ 3.87) shows a COSY correlation to H_2_-7 (a: δ_H_ 1.74; b: δ_H_ 1.21), and HMBC correlation with quaternary C-10 and the methine C-8 (δ_C_ 24.9). H-8 (δ_H_ 2.23) correlates in the HMBC with C-10, establishing a decalin ring system with the new cyclohexane ring fused to the cyclohexenone ring. A pendant methyl group (H_3_-19, δ_H_ 1.28) with HMBC correlations to C-1 and C-10 must be placed at the ring junction. H-8 further correlates in the HMBC with C-14 (δ_C_ 56.6) and C-11. COSY correlations between H-9 (δ_H_ 2.07) and H-11 (δ_H_ 5.02), then H-11 and H_2_-12 (a: δ_H_ 2.20; b: δ_H_ 1.48) support an extended branch from the decalin system that, taken with HMBC correlations for H_2_-12 to C-11, C-18 (δ_C_ 15.8)m and C-13 (δ_C_ 43.7), and H_3_-18 (δ_H_ 1.22) to C-13 and C-14 (δ_C_ 56.6), establishes a third ring fused to the previously established decalin. A fourth ring, the five-membered ring of a steroid ring system, was established by observation of a COSY correlation between H-14 (δ_H_ 1.31) and H-15 (δ_H_ 5.34), between H-15 and H-16 (δ_H_ 5.51), and between H-16 and H-17 (δ_H_ 1.34), all of which were HMBC correlated with C-13.

Left to assign were the steroid side chain and the acetate groups. The two ends of the steroid side chain were readily determined by HMBC correlations among the protons and carbons of positions 17, 20, 21, and 22, as well as 24, 25, and 26/27. Very weak correlations could be discerned between C-23 (δ_C_ 24.4) and H-22b (δ_H_ 0.90) and H-20 (δ_H_ 1.76), as well as H-23a (δ_H_ 1.36) and C-24 (δ_C_ 39.1), but overlapping and otherwise weak signals made assignments of C-23 to the rest of the well-established side chain challenging. The positions of the acetate groups were readily established by HMBC correlation of the oxymethine protons to the attached ester carbonyl; similarly, the acetate methyl groups could be positioned on their respective carbonyls (Figure 3).

**Figure 4:**
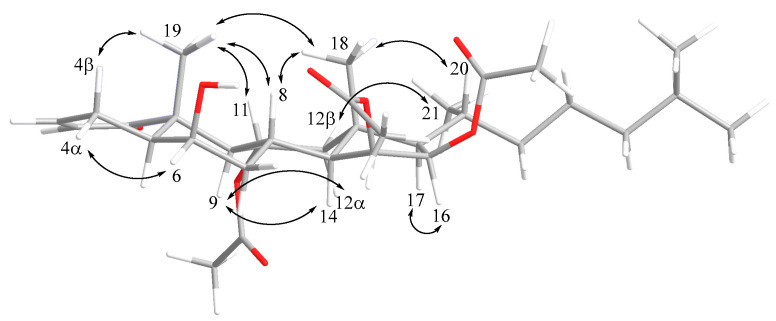
MM2 energy-minimized structure overlaid with ROESY relationships which established many of the relative configurational relationships of alcyosterone (**5**).

**Figure 5:**
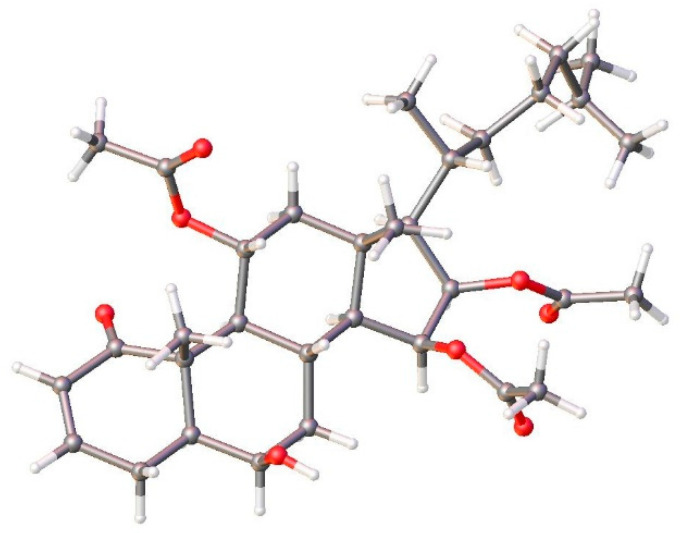
Asymmetric unit of **5** with anisotropic displacement parameters drawn at 50% probability level.

The stereochemical features of alcyosterone (**5**) were studied by ROESY and X-ray diffraction (XRD) analysis. Many of the relative relationships could be discerned in the ROESY spectrum (Figure 4), including methyl group H_3_-19 (δ_H_ 1.28), H-4β, H-8, and H-11 co-locating on the same face of the ring system and defining the A/B rings as a *trans*-decalin. Additional relationships were evident between H_3_-18, H-20, and H-8; H-12α and H_3_-21; H-9 and H-14; H-9 and H-12α; H-16 and H-17; H-15 and H-7β; and H-6 and H-4β (see Figure S22). These relationships were confirmed by XRD, which also provided the absolute stereochemistry (Figure 5).

Alcyopterosins are known to be mildly cytotoxic toward human tumor cell lines [16,19] but little attention has been focused on their infectious disease (ID) activity. Metabolites from *Alcyonium* sp. indet. isolated in this study in sufficient quantity were therefore screened in three ID assays. Alcyopterosins V (**3**), E (**4**), and alcyosterone (**5**) were inactive against the ESKAPE panel of bacterial pathogens, but both **3** and **4** demonstrated potent activity against *Clostridium difficile*, a difficult-to-treat intestinal bacterium which afflicts up to half a million people annually and caused 30,000 deaths in 2015 [20]. Alcyopterosin E (MIC 6.9 μM) was slightly more active against *C. difficile* than alcyopterosin V (MIC 8.1 μM). Cytotoxicity against host cell lines HEK293T and HepG2 also found **4** less toxic (CC_50_ 570 and 331 μM, respectively) than **3** (CC_50_ 220 and 288 μM, respectively). Vancomycin as a control displays an MIC of 0.34 μM against *C. difficile* and was non-toxic to the host cells at the same concentrations alcyopterosins were assayed.

Alcyopterosin C, E (**4**), L, 4,12-bis(acetyl)alcyopterosin O, V (**3**), and alcyosterone (**5**) were screened against *Leishmania donovani* and found with roughly equal, single digit μM, activity [21]. Leishmania, the disease caused by *L. donovani*, is often disfiguring and can lead to death if not properly treated, though current treatment regimes can be expensive and toxic, and are considered ineffective [22]. The highest potency was displayed by 4,12-Bis(acetyl)alcyopterosin O (IC_50_ 1.2 μM), though alcyosterone (IC_50_ 1.5 μM), alcyopterosin L (IC_50_ 2.4 μM), and alcyopterosin E (IC_50_ 3.1 μM) were largely indistinguishable. Alcyopterosin V (IC_50_ 7.0 μM) and alcyopterosin C (IC_50_ 13 μM) were only slightly less potent than the control, miltefosine (IC_50_ 6.2 μM). Only **3** and **4** were available in sufficient quantity to assay against the *Leishmania* host cell line, J774.A1 macrophages, which showed alcyopterosin E, though low in toxicity, was twice as toxic (IC_50_ 62 μM) as alcyopterosin V (IC_50_ 110 μM) to the mammalian cells.

## 3. Materials and Methods

### 3.1. General Experimental Procedures

Optical rotations were measured on a Rudolph Research Analytical AUTOPOL IV digital polarimeter at 589 nm. UV absorptions were acquired with an Agilent Cary 60 UV-vis spectrophotometer. IR spectra were recorded with an Agilent Cary FTIR 630 spectrometer and PerkinElmer Spectrum Two equipped with a UATR (single reflection diamond) sample introduction system. NMR spectra were recorded on Varian Direct Drive 500 MHz and Varian Inova 500 MHz spectrometers. Chemical shifts are reported with the use of the residual CDCl_3_ signals (*δ*_H_ 7.27 ppm; *δ*_C_ 77.0 ppm) as internal standards for ^1^H and ^13^C NMR spectra, respectively. COSY, HSQC, HMBC, and ROESY experiments corroborated the ^1^H and ^13^C NMR assignments. Analytical LC/MS with a Phenomenex Kinetex C18 column (50 × 2.1 mm, 2.6 μm) on an Agilent 6230 LC/TOF-MS with electrospray ionization detection provided the high-resolution masses. Semi-preparative and analytical HPLC separations were performed on a Shimadzu LC-20 AT system equipped with an ultraviolet (UV) detector using a Luna silica column (5 μm, 250 × 10 mm), and a YMC C-18 column (10 μm, 150 × 4 mm). MPLC was performed on a Teledyne Isco CombiFlash Rf 200i equipped with an evaporative light-scattering detector (ELSD) and a multiwavelength UV detector using a RediSep Rf silica 80 g flash column, and silica gel 230–400 mesh was used to load samples.

### 3.2. Biological Material

The soft coral was collected via trawling on the *R/V* Nathaniel B. Palmer vessel during the austral autumn in late April 2013. The specimens were collected between 126 and 130 m depth, frozen immediately upon collection, and maintained at −80 °C until extraction. The tissue of the frozen specimens was subsampled and preserved in 96% ethanol. Subsequent extraction was performed using a DNeasy blood and tissue kit (Qiagen) following manufacturer’s protocols. Using primers ND42599F/mut3458R [23,24], a piece of the mitochondrial genome was amplified (*msh1*, a homolog of *mutS*). Cycling conditions included an initial 5× cycles at 45 °C annealing, followed by 39× cycles at 58 °C. Amplicons were sent to the Australian Genome Research Facility, Perth for purification and Sangar sequencing. The resulting bi-directional sequence was assembled and edited, primers removed, deposited in GenBank (OP429120), and aligned with other soft coral sequences from GenBank. A Maximum-Likelihood analysis using IQ-tree [25], implementing the evolutionary model VM+F+G4 selected with ModelFinder [26], was carried out. The nodes were tested with 1000 ultrafast bootstrap replicates.

### 3.3. Extraction and Isolation of Coral Metabolites

The frozen soft coral was freeze-dried, and 420 g of dry weight material was extracted using a 1:1 ratio of dichloromethane/methanol, three times over 3 days. The extract was dried, and the yielded 25.0 g were resolubilized in ethyl acetate and partitioned against H_2_O. The concentrated EtOAc partition fraction (11.4 g) was resuspended in EtOAc and dried onto silica gel for fractionation by MPLC on a Teledyne CombiFlash fitted with UV and ELS detection. Fractions A through I eluted from MPLC using ethyl acetate/*n*-hexanes (0:100) to ethyl acetate/*n*-hexanes (100:0) over 25 min. Fractions D through H displayed NMR signature signals of marine illudalane compounds, in particular the aromatic singlet (H-8) and a midfield oxymethylene (H_2_-4), and were selected for purification using normal-phase and reversed-phase HPLC with UV detection. Semi-preparative NP HPLC using *n*-hexane to EtOAc/*n*-hexanes (1:1) over 25 min gradient, yielded the known alcyopterosins C (**6**), G (**7**), and 4,12-bis(acetyl)alcyopterosin O (**8**) from MPLC fraction F. Alcyopterosin L (**9**) and newly isolated as natural product alcyopterosin V (**3**) (4.0 mg) came from MPLC fraction H. Alcyopterosin E (**4**) was derived from fraction G. New alcyopterosins T (**1**) (0.5 mg) and U (**2**) (0.5 mg) came from fraction E, along with 4,12-bis(acetyl)alcyopterosin O (1.6 mg) and alcyopterosins C (2.0 mg), E (7.5 mg), G (0.6 mg), and L (1.4 mg).

Soxhlet extraction of an additional specimen in dichloromethane followed by a similar chromatographic profile described above resulted in seven fractions. Further purification of fraction E, via normal phase HPLC with a hexane–ethyl acetate (1:1) gradient, followed by reversed-phase HPLC using a water–acetonitrile (70% to 100%) gradient, led to alcyosterone (**5**) (1.2 mg).

*Alcyopterosin T* (**1**): colorless oil; UV (CH_2_Cl_2_) λ_max_ (log ε): 225 (1.52), 245 (1.45), 340 (1.24) nm; IR ν_max_: 3000, 2900, 2850, 1720, 1640, 1600, 1280 cm^−1^; for ^1^H and ^13^C NMR data see Table 1; HRESIMS [M + Na]^+^: *m*/*z* 344.1460 (calcd for C_17_H_23_NO_5_Na, *m*/*z* 344.1468).

*Alcyopterosin U* (**2**): colorless oil; UV (CH_2_Cl_2_) λ_max_ (log ε): 225 (1.76), 230 (1.59), 250 (1.55), 264 (1.54), 305 (1.52), 330 (1.47), 365 (1.44) nm; IR ν_max_: 3000, 2900, 2850, 1750, 1700, 1640, 1600, 1280 cm^−1^; for ^1^H and ^13^C NMR data see Table 1; HRESIMS [M + H]^+^: *m*/*z* 336.1429 (calcd for C_17_H_22_NO_6_, *m*/*z* 336.1442).

*Alcyopterosin V* (**3**): for ^1^H and ^13^C NMR data see Table 1. HRESIMS [M + H]^+^: *m*/*z* 247.1328 (Calcd for C_15_H_19_O_3_, 247.1329).

*Alcyosterone* (**5**): translucent solid; [α]^24.6^_365_ -125° (*c* 2 × 10^−3^ g/mL, ACN); UV (ACN) λ_max_ (log ε): 215 (2.60), 235 (2.68) nm; IR υ_max_: 1250, 1690, 1700, 1750, 2850, 2900, 2950 cm^−1^; for ^1^H and ^13^C NMR data see Table 2; HRESIMS [M + H]^+^: *m*/*z* 575.3555 (calcd for C_33_H_50_O_8_H, *m*/*z* 575.3578); [M − OAc]^+^ *m*/*z* 515.3364 (calcd for C_31_H_47_O_6_, *m*/*z* 515.3367).

### 3.4. Leishmania donovani Infected Macrophage Assay

The *Leishmania donovani* infected macrophage assay and cytotoxicity screen were conducted as previously described [27].

### 3.5. Clostridium difficile Susceptibility Screening

The screening against *C. difficile* was performed in two steps. In step 1, overnight culture of a hyper-virulent clinical strain *C. difficile* UK6 was inoculated into a fresh BHIS medium at a volume ratio of 1:1000. After pre-incubation at 37 °C under an anaerobic atmosphere for 2 h, the bacterial culture was divided into a sterile 96-well plate and each well contained 192 μL of bacterial culture. Then, 8 μL of each extract was added to each well of the plate, mixed thoroughly, and incubated at 37 °C in an anaerobic chamber for 48 h. Control groups of 200 μL of BHIS medium only, 200 μL of bacterial culture only, and 192 μL of bacterial culture in 8 μL of DMSO were also included in separate columns within each plate. Extracts that displayed initial antibacterial activity were further evaluated for their minimum inhibitory concentration (MIC) against *C. difficile*. Serial dilutions of each extract (400 μg/mL, 200 μg/mL, 100 μg/mL, 50 μg/mL, 20 μg/mL, 10 μg/mL, 5 μg/mL, and 2 μg/mL) were prepared in a fresh BHIS medium. Then, 100 μL of each extract dilution was added to 100 μL of bacterial culture (pretreated as described), mixed well, and incubated at 37 °C in an anaerobic chamber for 48 h. Control groups including wells containing fresh medium only and bacterial culture only were also included as described. Activity was determined as +/− (clear or turbid (OD_600_) culture). The MICs of the three recommended antibiotics metronidazole, vancomycin, and fidaxomicin against *C. difficile* UK6 were also determined using broth microdilution methodology.

### 3.6. Determination of the Half Maximal Inhibitory Concentration (IC_50_) toward Human Liver Cells and Kidney Cells

The cytotoxicity of the metabolites to human liver cells and kidney cells was determined using an MTT based-In Vitro Toxicology Assay Kit (Sigma–Aldrich, St. Louis, MO, USA) following the manufacturing instructions. The human kidney HEK293T cells and the human liver HEPGZ cells were used for the evaluation in this study. Both cell samples were maintained and suspended in Dulbecco’s Modified Eagle Medium (DMEM with 4.5 g/L glucose, L-glutamine and sodium pyruvate, Corning, Manassas, VA, USA) containing 10% fetal bovine serum (Thermo Scientific) and 1% penicillin/streptomycin at 37 °C under 5% CO_2_ atmosphere. The cells were plated on a 96-well plate with approximately 5 × 10^3^–1 × 10^4^ cells in each well, and incubated at 37 °C overnight. After that, each of the selected extracts from the antimicrobial susceptibility test was added to the wells and incubated with the cells at a series of 2-fold diluted concentrations ranging from 128 μg/mL to 0.125 μg/mL. Following a 24 h of incubation, 10 μL of 1-(4,5-dimethylthiazol-2-yl)-3,5-diphenylformazan (MTT) stock solution (5 mg/mL) was added to each well of the cells, mixed well, and incubated at 37 °C for another 4 h. After that, the liquid in each well of the plate was removed carefully and thoroughly, then the cells in the wells were treated with 100 μL of DMSO, and incubated at 37 °C for 15 m. Optical density (OD) values were measured at a wavelength of 540 nm (OD_540_) using a microplate reader (Synergy HTX; Bio Tek Instruments, Inc. Winooski VT). Cells treated with vancomycin, a common option for treating CDI in clinical settings, were also included in the MTT tests as a control. Cell survival and the IC_50_ were calculated according to the method used in a previous publication [26]: Survival of cells (%) = Drug-treated group OD_540_/control group OD_540_ × 100. The IC_50_ value was calculated as follows: lgIC_50_ = X_m_ − I [P − (3 − P_m_ − P_n_)/4], where X_m_ was the log maximum dose, I was the log (maximum dose/adjacent dose), P was the sum of the positive response rate, P_m_ was the maximum positive response rate, and P_n_ was the minimum positive response rate.

### 3.7. X-ray Diffraction of Alcyosterone (***5***)

XRD methodology was conducted as we have previously done [28]. Data and refinement conditions are shown in Table S2. CCDC Deposition Number 2205919.

## 4. Patents

US patent 10,898,460, *Leishmania* Inhibitors, based on portions of this work was awarded 26 January 2021.

## Figures and Tables

**Table 1:** ^1^H and ^13^C NMR Data for Alcyopterosins T, U, and V (**1**–**3**).

Position	Alcyopterosin T (1)		Alcyopterosin U (2)		Alcyopterosin V (3)	
	δ_C_, ^1^ Type	δ_H_, ^2^ Mult. (*J*)	δ_C_, ^1^ Type	δ_H_, ^2^ Mult. (*J*)	δ_C_, ^1^ Type	δ_H_, ^2^ Mult. (*J*)
1	47.1, CH_2_	2.79, s	42.2, CH_2_	3.03, s	44.8, CH_2_	3.04, s
2	143.2, C		151.5, C		146.7, C	
3	131.1, C		133.0, C		122.5, C	
4	72.5, CH_2_	4.57, t (7.9)	71.9, CH_2_	4.60, t (7.6)	63.2, CH_2_	4.25, dd (2.5, 12.6)
						3.81, dd (6.1, 12.6)
5	27.7, CH_2_	3.15, t (7.9)	28.6, CH_2_	3.28, t (7.7)	82.2, CH	5.55, br dd (2.0, 5.9)
6	131.4, C		142.2, C		142.4, C	
7	135.9, C		138.2, C		130.0, C	
8	128.0, CH	7.06, s	127.4, CH	7.64, s	131.8, CH	7.24, s
9	143.4, C		135.4, C		141.1, C	
10	48.5, CH_2_	2.73, s	211.4, C		47.0, CH_2_	2.74, s
11	40.4, C		46.3, C		40.9, C	
12	62.5, CH_2_	5.16, s	61.0, CH_2_	5.25, s	170.8, C	
13	20.7, CH_3_	2.38, s	21.0, CH_3_	2.47, s	18.0, CH_3_	2.37, s
14	29.7, CH_3_	1.17, s	26.2, CH_3_	1.24, s	28.8, CH_3_	1.16, s
15	29.7, CH_3_	1.17, s	26.2, CH_3_	1.24, s	28.8, CH_3_	1.19, s
1′	171.1, C		171.2, C			
2′	21.7, CH_3_	2.09, s	21.8, CH_3_	2.10, s		

^1^ CDCl_3_, 200 MHz, shift, and type determined from HSQC and HMBC; ^2^ CDCl_3_, 600 MHz, *J* in Hz.

**Table 2:** ^1^H and ^13^C NMR Spectroscopic Data for Alcyosterone (**5**).

Position	*δ*_C_^1^, Type	*δ*_H_, ^2^ Integ., Mult., *J*
1	203.9, C	
2	128.4, CH	5.83, 1H, dd, 2.2, 9.9
3	142.5, CH	6.58, 1H, ddd, 2.1, 4.8, 9.6
4	28.4, CH_2_	2.79, 1H, dddd, 0.7, 2.4, 11.4, 19.8
		2.11, 1H, ddd, 0.8, 4.8, 19.5
5	46.6, CH	1.86, 1H, ddd, 0.7, 2.9, 10.8
6	69.7, CH	3.87, 1H, q, 2.4
7	36.8, CH_2_	1.74, 1H, ov ^3^
		1.21, 1H, ov
8	24.9, CH	2.23, 1H, ov
9	47.8, CH	2.07, 1H, ov
10	47.7, C	
11	70.4 *, C	5.02, 1H, dt, 3.9, 11
12	46.7, CH_2_	2.20, 1H, ov
		1.48, 1H, ov
13	43.7, CH	
14	56.6, CH	1.31, 1H, dd, 5.8, 11.2
15	70.5 *, CH	5.34, 1H, dd, 6.3, 6.6
16	73.0, CH	5.51, 1H, dd, 6.9, 7.0
17	59.9, CH	1.34, 1H, ov
18	15.8, CH_3_	1.22, 3H, s
19	13.2, CH_3_	1.28, 3H, s
20	30.0, CH	1.76, 1H, ov
21	18.2, CH_3_	0.95, 3H, d, 6.6
22	35.6, CH_2_	1.20, 1H, ov
		0.90, 1H, ov
23	24.4, CH_2_	1.36, 1H, ov
		1.11, 1H, ov
24	39.1, CH_2_	1.09, 1H, ov
		1.05, 1H, d, 6.6
25	27.9, CH	1.48, 1H, ov
26	22.6, CH_3_	0.85, 3H, d, 6.5
27	22.4, CH_3_	0.85, 3H, d, 6.5
1′	170.4, C	
2′	21.5, CH_3_	1.93, 3H, s
3′	169.9, C	
4′	20.7, CH_3_	2.06, 3H, s
5′	169.4, C	
6′	20.5, CH_3_	2.02, 3H, s

^1^ CDCl_3_, 125 MHz, type determined from HSQC; ^2^ CDCl_3_, 500 MHz, *J* in Hz. ^3^ ov = overlapping signal. * Interchangeable

## Data Availability

The mutS gene sequence was deposited with Genebank (accession number OP429120). X-ray crystallographic data was deposited with the Cambridge Crystallographic Data Center (Deposition Number 2205919).

## Supplementary Materials

The following supporting information can be downloaded at: https://www.mdpi.com/article/10.3390/md20090576/s1, Figure S1. Maximum Likelihood tree topology comparing *msh1* sequences of our *Alcyonium* specimen with those available in GenBank; Figure S2. ^1^H NMR spectrum of alcyopterosin T (**1**), 500 MHz, CDCl_3_; Figure S3. COSY spectrum of alcyopterosin T (**1**), 500 MHz, CDCl_3_; Figure S4. HSQC spectrum of alcyopterosin T (**1**), 500 MHz, CDCl_3_; Figure S5. HMBC spectrum of alcyopterosin T (**1**), 500 MHz, CDCl_3_; Figure S6. HRESIMS of alcyopterosin T (**1**); Figure S7. ^1^H NMR spectrum of alcyopterosin U (**2**), 500 MHz, CDCl_3_; Figure S8. COSY spectrum of alcyopterosin U (**2**), 500 MHz, CDCl_3_; Figure S9. HSQC spectrum of alcyopterosin U (**2**), 500 MHz, CDCl_3_; Figure S10. HMBC spectrum of alcyopterosin U (**2**), 500 MHz, CDCl_3_; Figure S11. HRESIMS of alcyopterosin U (**2**). Calculated for C17H21NO6H, 336.1442; Figure S12. ^1^H NMR spectrum of alcyopterosin V (**3**), 500 MHz, CDCl_3_; Figure S13. ^13^C NMR spectrum of alcyopterosin V (**3**), 125 MHz, CDCl_3_; Figure S14. COSY spectrum of alcyopterosin V (**3**), 500 MHz, CDCl_3_; Figure S15. HSQC spectrum of alcyopterosin V (**3**), 500 MHz, CDCl_3_; Figure S16. HMBC spectrum of alcyopterosin V (**3**), 500 MHz, CDCl_3_; Figure S17. HRESIMS of alcyopterosin V (**3**); Figure S18. ^1^H NMR spectrum of alcyosterone (**5**), 500 MHz, CDCl_3_; Figure S19. ^13^C NMR spectrum of alcyosterone V (**5**), 125 MHz, CDCl_3_; Figure S20. COSY spectrum of alcyosterone (**5**), 500 MHz, CDCl_3_; Figure S21. HSQC spectrum of alcyosterone (**5**), 500 MHz, CDCl_3_; Figure S22. HMBC spectrum of alcyosterone (**5**), 500 MHz, CDCl_3_; Figure S23. HRESIMS of alcyosterone (**5**); Table S1. NMR shift comparison between compounds isolated in the current work to those previously published; Table S2. Crystal data and structure refinement for alcyosterone (**5**); Figure S24. Asymmetric unit of alcyosterone (**5**).
