# Supplementary material for: Chemistry and Bioactivity of the Deep-Water Antarctic Octocoral Alcyonium sp
Source: Mar Drugs. 2022 Sep 14;20(9):576. doi: 10.3390/md20090576 (PMC9505732; doi:10.3390/md20090576)
Supplement: Supplementary file 1 [file marinedrugs-20-00576-s001.zip › marinedrugs-1852865-supplementary.pdf]

marine drugs

MDPI

Supplementary Information

Chemistry and Bioactivity of the Deep-water Antarctic Coral *Alcyonium* sp.

Anne-Claire D. Limon [1,†], Hiran M. L. W. Patabendige [2], Ala Azhari [3], Xingmin Sun [2], Dennis E. Kyle [3,‡], Nerida G. Wilson [4] and Bill J. Baker [1,*]

[1] Department of Chemistry, University of South Florida, 4202 E. Fowler Avenue, CHE 205, Tampa, FL 33620, USA
[2] Department of Molecular Medicine, Morsani College of Medicine, University of South Florida, 12901 Bruce B. Downs Boulevard, MDC07, Tampa, FL 33612, USA
[3] USF Center for Global Health and Infectious Diseases Research, University of South Florida, 3010 USF Banyan Circle, IDRB 304, Tampa, FL 33612, USA
[4] Collections & Research, Western Australia Museum, 49 Kew Street, Welshpool 6106, Perth and University of Western Australia, 35 Stirling Highway, Crawley 6009, Australia
* Correspondence: bjbaker@usf.edu; Tel.: +1-813-974-1967
† Current address: Department of Chemistry, University of Southern Indiana, 8600 University Boulevard, SC 2255, Evansville, IN 47712, USA.
‡ Current address: Center for Tropical and Emerging Global Diseases, University of Georgia, Athens, GA 30602, USA.

Table of Contents

Figure S1. Maximum Likelihood tree topology comparing *msh1* sequences of our *Alcyonium* specimen with those available on Genbank........2
Figure S2. ¹H NMR spectrum of alcyopterosin T (**1**), 500 MHz, $CDCl_3$........3
Figure S3. COSY spectrum of alcyopterosin T (**1**), 500 MHz, $CDCl_3$........3
Figure S4. HSQC spectrum of alcyopterosin T (**1**), 500 MHz, $CDCl_3$........4
Figure S5. HMBC spectrum of alcyopterosin T (**1**), 500 MHz, $CDCl_3$........4
Figure S6. HRESIMS of alcyopterosin T (**1**)........5
Figure S7. ¹H NMR spectrum of alcyopterosin U (**2**), 500 MHz, $CDCl_3$........5
Figure S8. COSY spectrum of alcyopterosin U (**2**), 500 MHz, $CDCl_3$........6
Figure S9. HSQC spectrum of alcyopterosin U (**2**), 500 MHz, $CDCl_3$........6
Figure S10. HMBC spectrum of alcyopterosin U (**2**), 500 MHz, $CDCl_3$........7
Figure S11. HRESIMS of alcyopterosin U (**2**). Calculated for C17H21NO6H, 336.1442........7

Figure S12. ^{1}H NMR spectrum of alcyopterosin V (**3**), 500 MHz, $CDCl_3$..........8
Figure S13. ^{13}C NMR spectrum of alcyopterosin V (**3**), 125 MHz, $CDCl_3$..........8
Figure S14. COSY spectrum of alcyopterosin V (**3**), 500 MHz, $CDCl_3$..........9
Figure S15. HSQC spectrum of alcyopterosin V (**3**), 500 MHz, $CDCl_3$..........9
Figure S16. HMBC spectrum of alcyopterosin V (**3**), 500 MHz, $CDCl_3$..........10
Figure S17. HRESIMS of alcyopterosin V (**3**)..........10
Figure S18. ^{1}H NMR spectrum of alcyosterone (**5**), 500 MHz, $CDCl_3$..........11
Figure S19. ^{13}C NMR spectrum of alcyosterone (**5**), 125 MHz, $CDCl_3$..........11
Figure S20. COSY spectrum of alcyosterone (**5**), 500 MHz, $CDCl_3$..........12
Figure S21. HSQC spectrum of alcyosterone (**5**), 500 MHz, $CDCl_3$..........12
Figure S22. HMBC spectrum of alcyosterone (**5**), 500 MHz, $CDCl_3$..........13
Figure S23. HRESIMS of alcyosterone (**5**)..........13
Table S1. NMR shift comparison between compounds isolated in the current work to those previously published..........14
Table S2. Crystal data and structure refinement for alcyosterone (**5**)..........15
Figure S24. Asymmetric unit of alcyosterone (**5**) 15

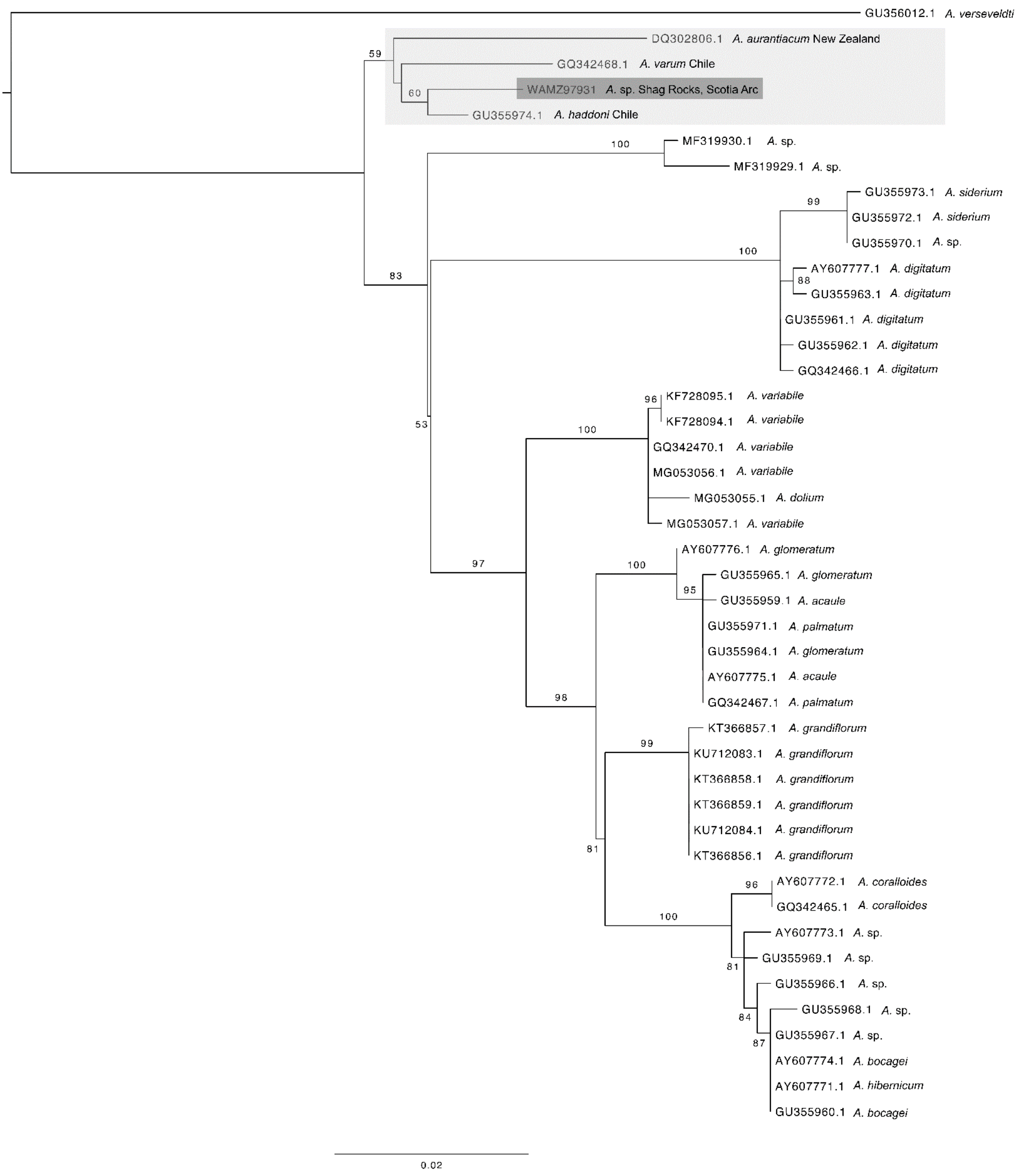

Figure S1. Maximum Likelihood tree topology comparing *msh1* sequences of *Alcyonium* specimen with those available on Genbank.

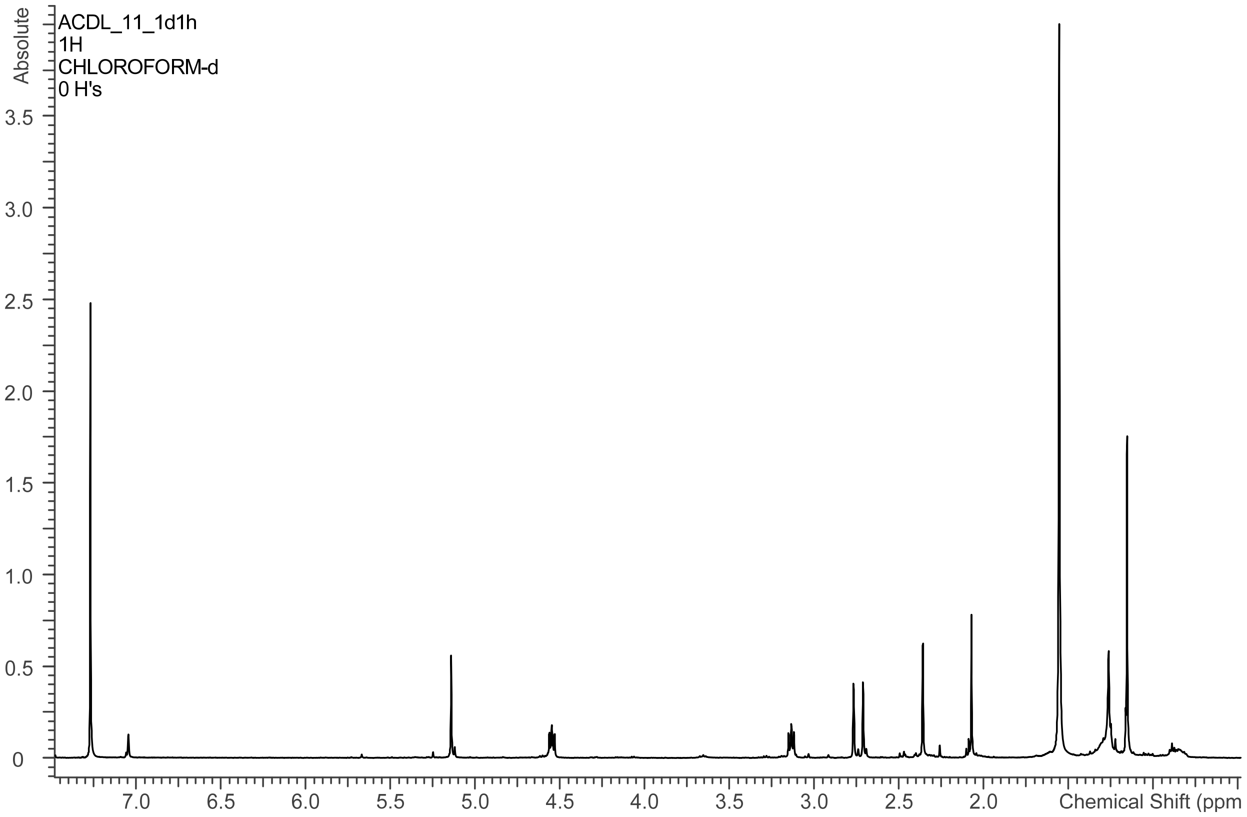

Figure S2. ^{1}H NMR spectrum of alcyopterosin T (**1**), 500 MHz, $CDCl_3$.

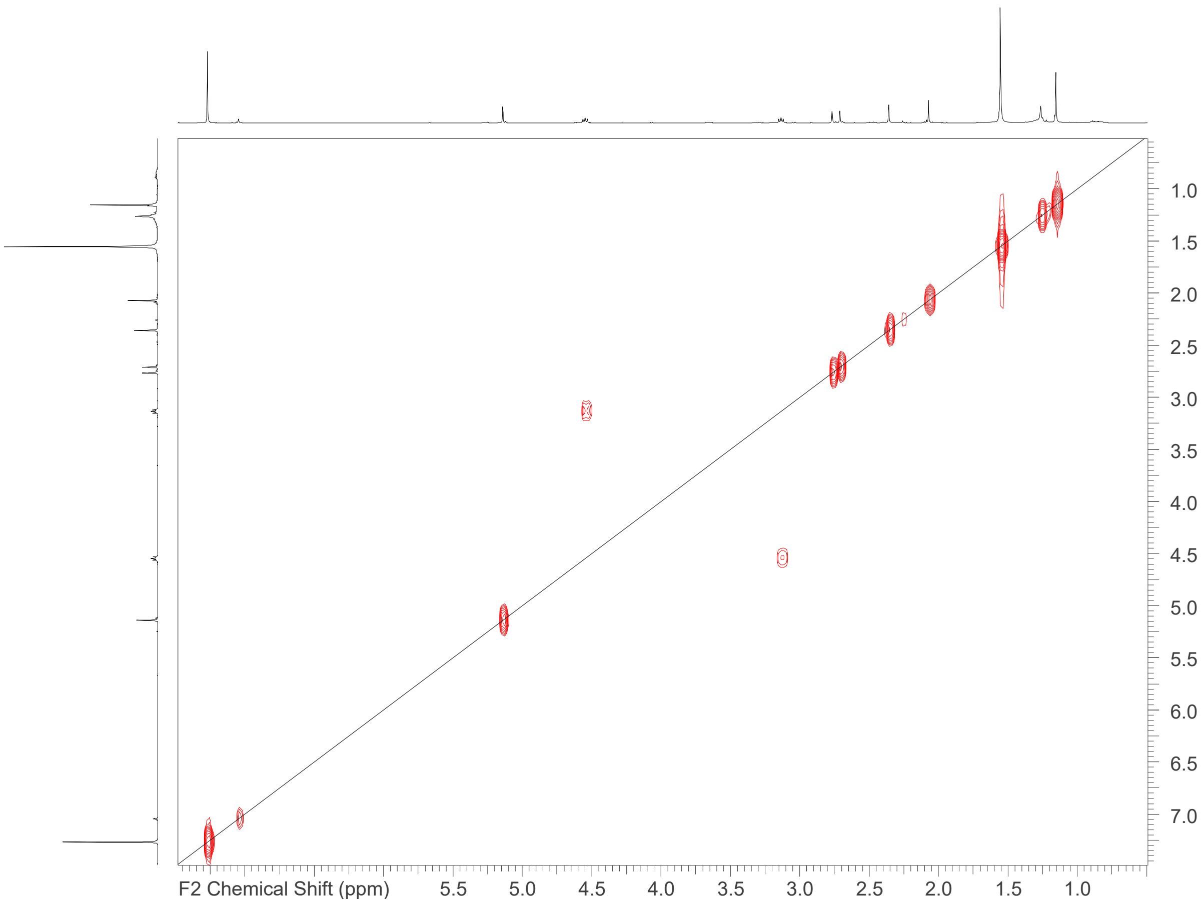

Figure S3. COSY spectrum of alcyopterosin T (**1**), 500 MHz, $CDCl_3$.

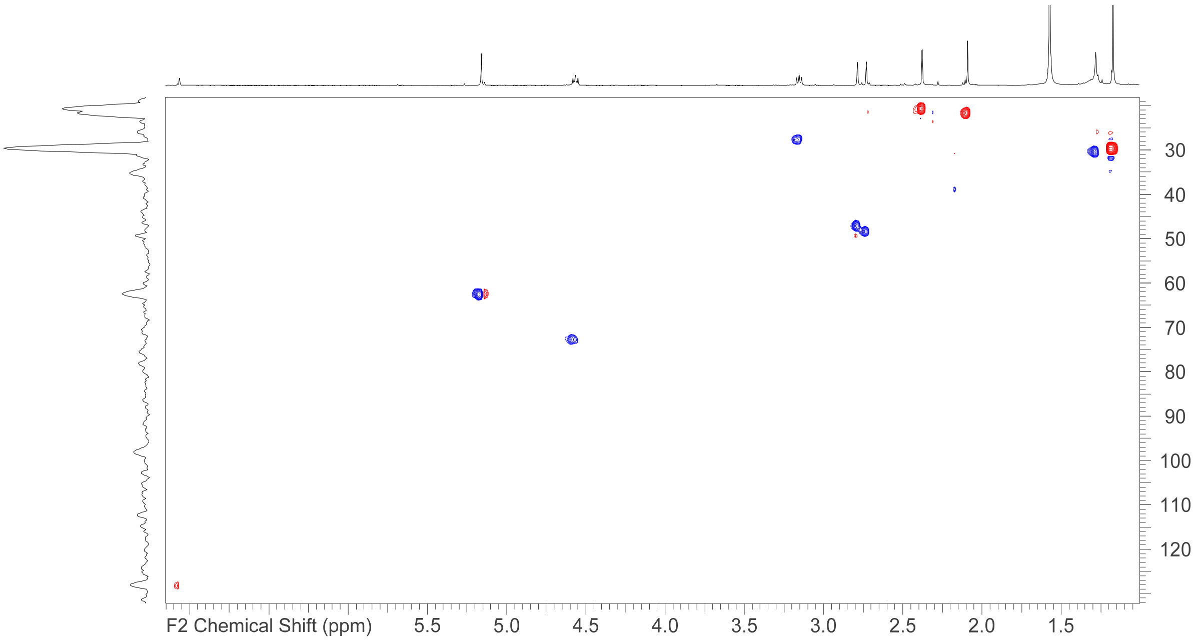

Figure S4. HSQC spectrum of alcyopterosin T (**1**), 500 MHz, $CDCl_3$.

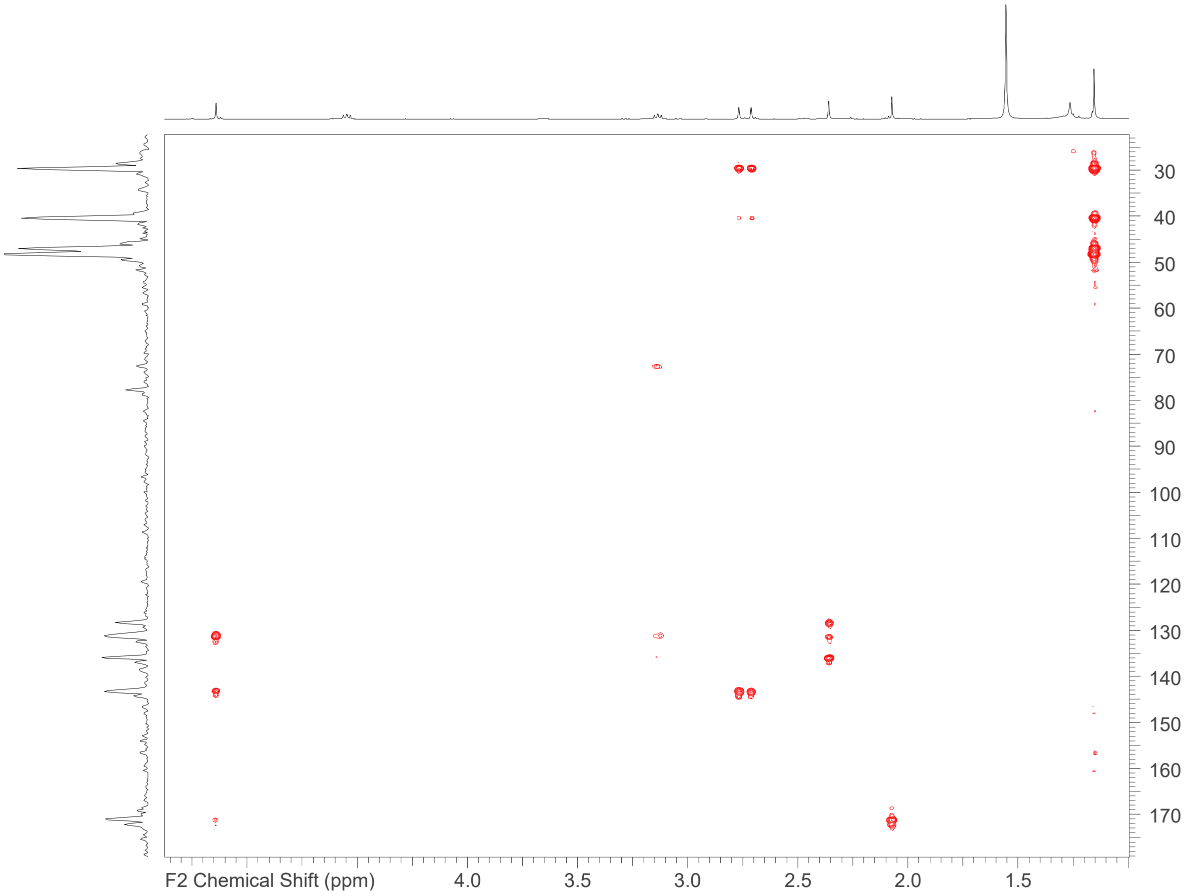

Figure S5. HMBC spectrum of alcyopterosin T (**1**), 500 MHz, $CDCl_3$.

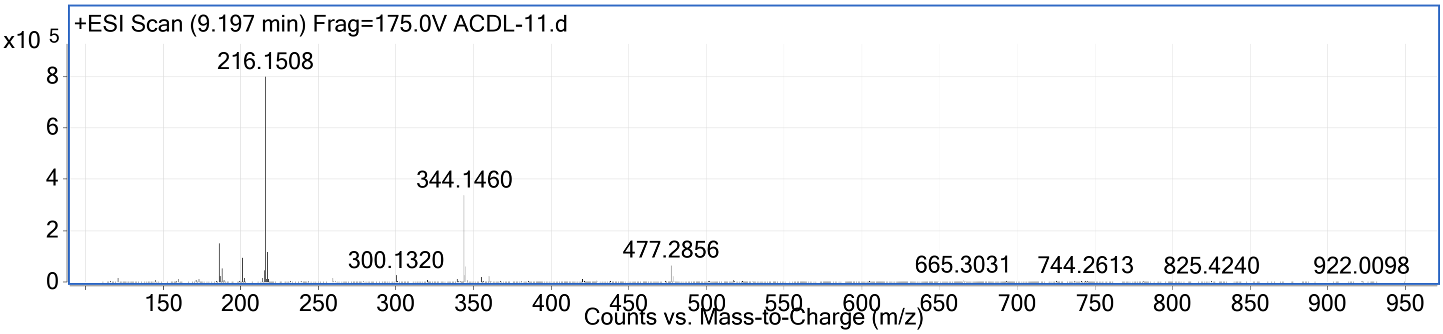

Figure S6. HRESIMS of alcyopterosin T (**1**). Calculated for $C_{17}H_{23}NO_5Na$, 344.1468.

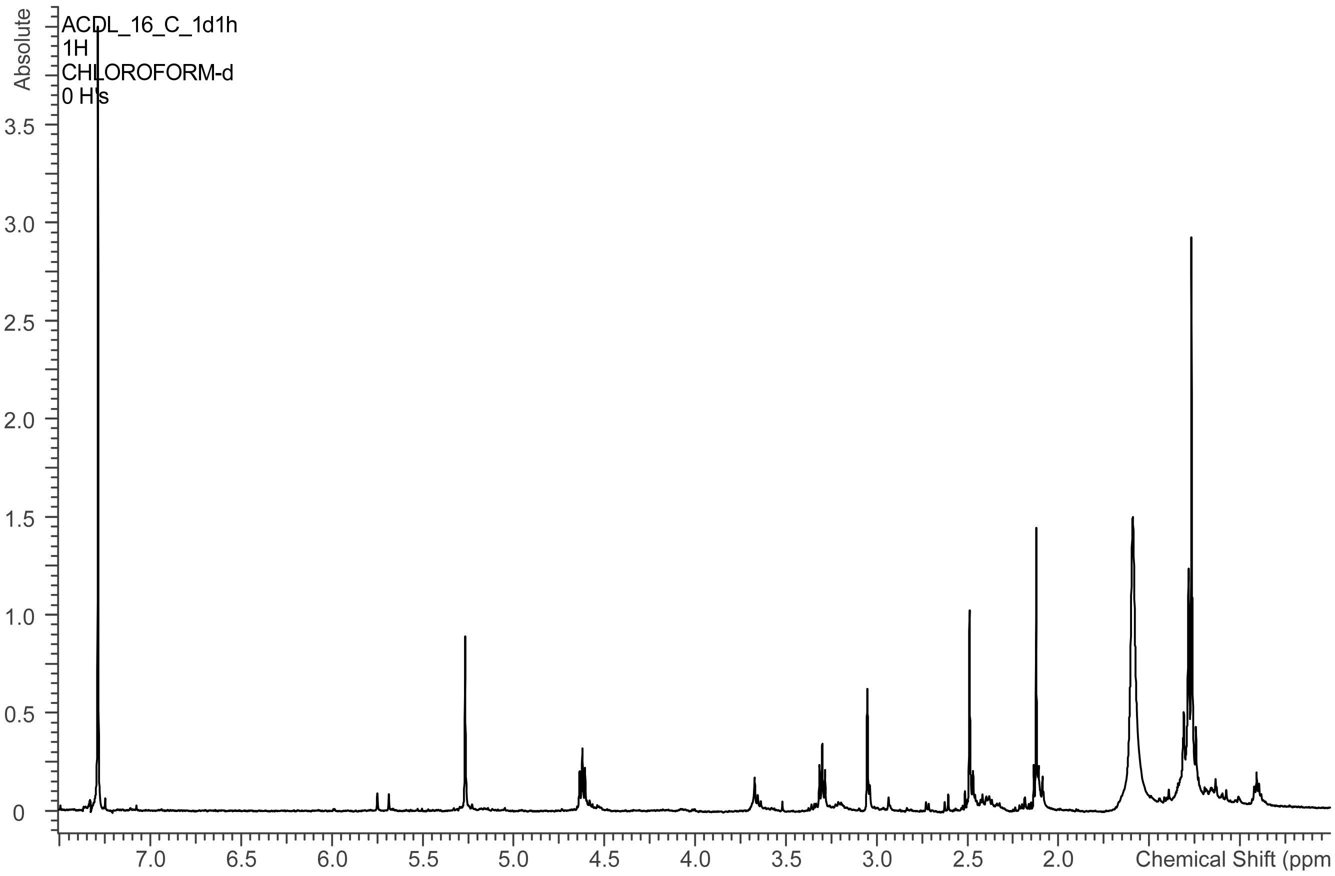

Figure S7. ^{1}H NMR spectrum of alcyopterosin U (**2**), 500 MHz, $CDCl_3$.

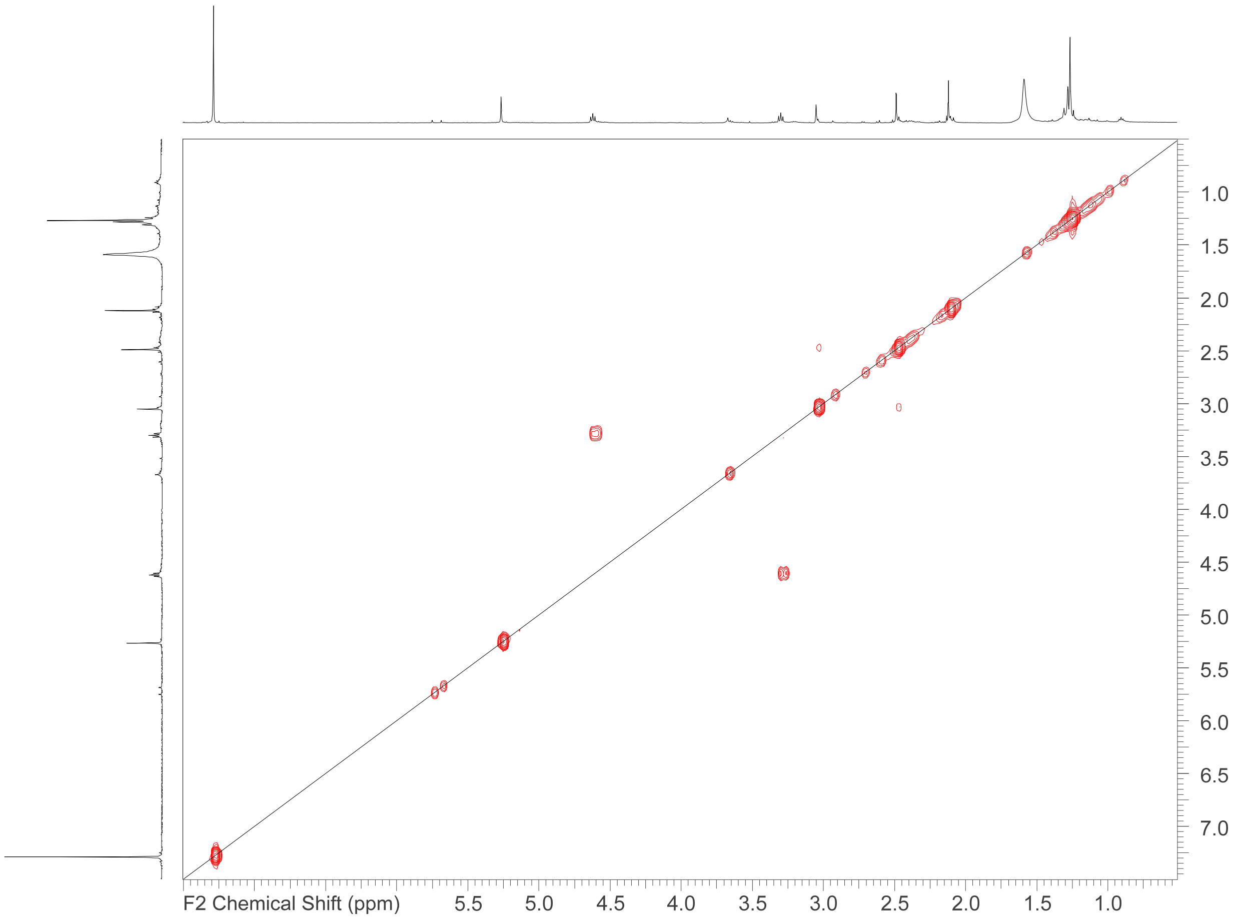

Figure S8. COSY spectrum of alcyopterosin U (**2**), 500 MHz, $CDCl_3$.

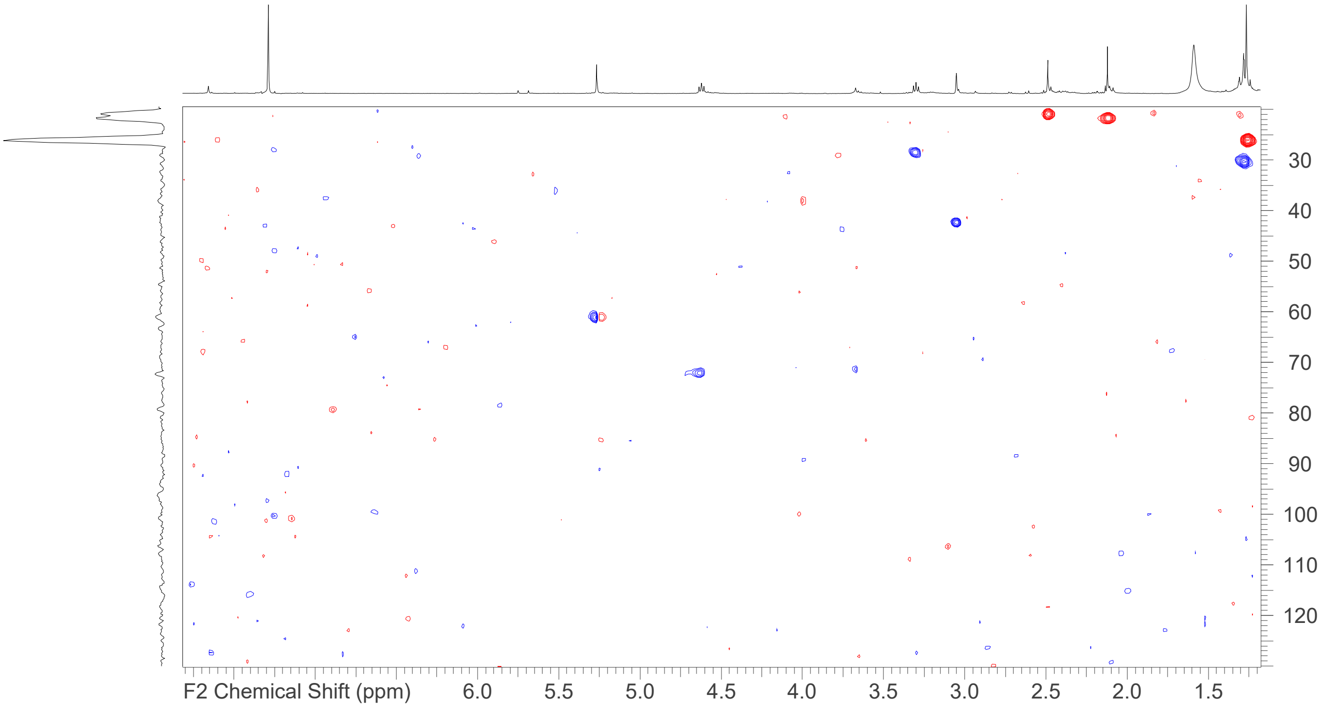

Figure S9. HSQC spectrum of alcyopterosin U (**2**), 500 MHz, $CDCl_3$.

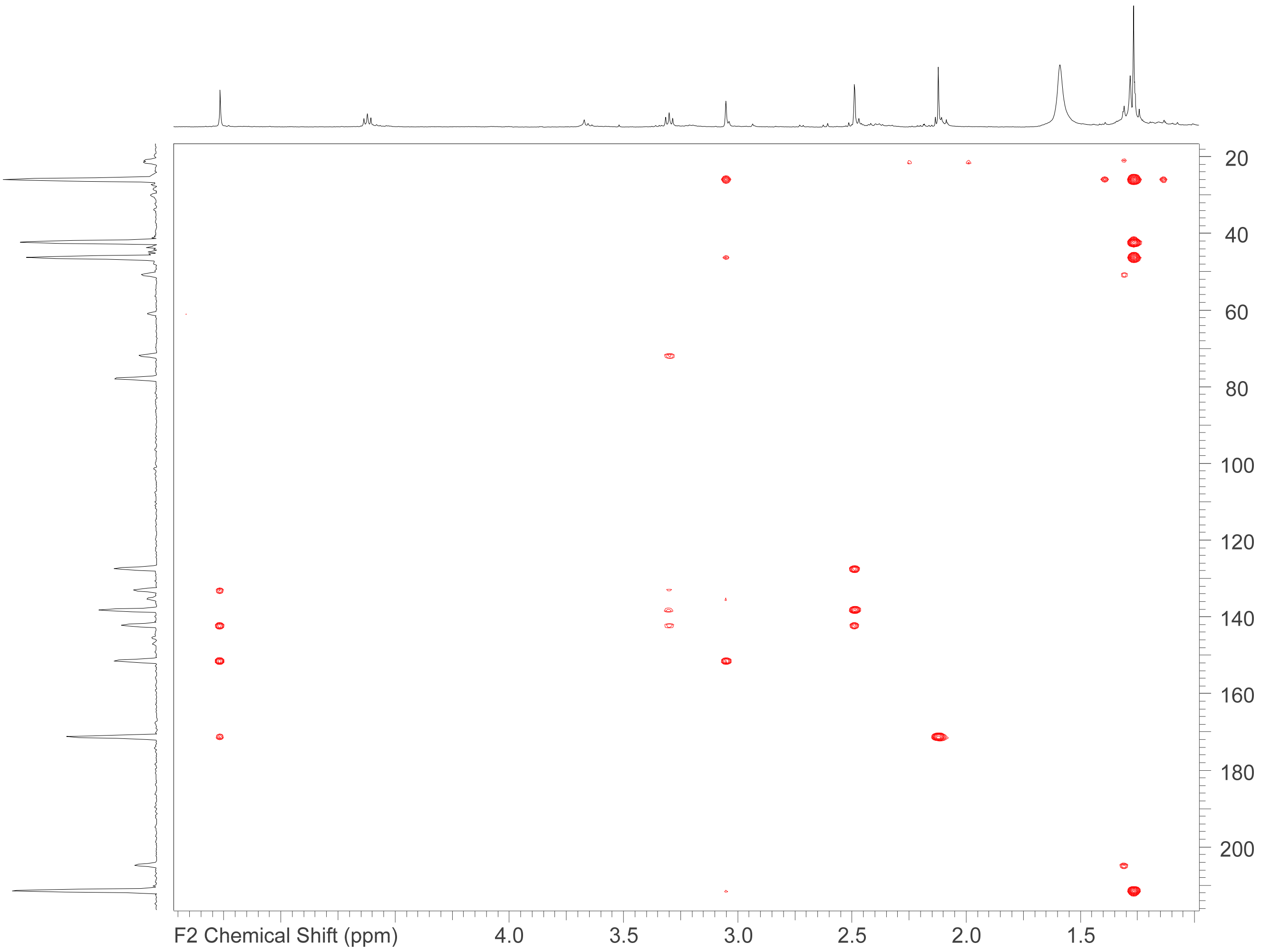

Figure S10. HMBC spectrum of alcyopterosin U (**2**), 500 MHz, $CDCl_3$.

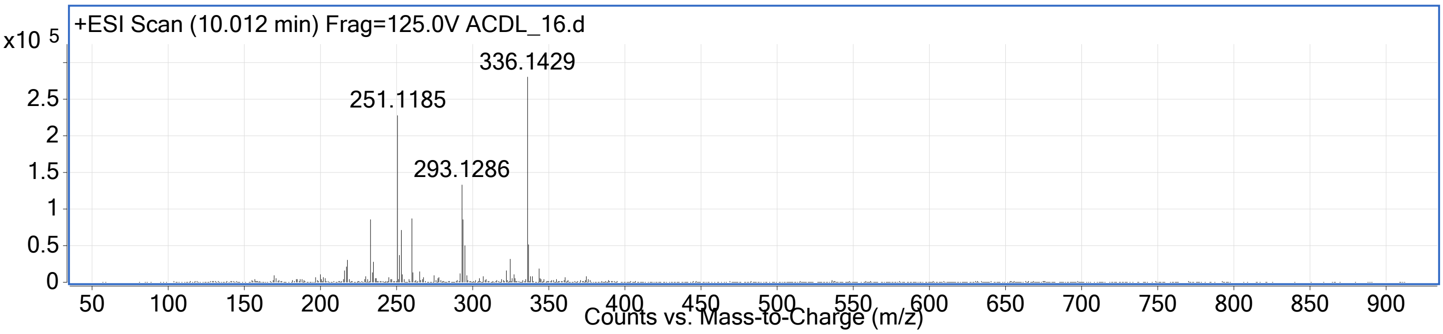

Figure S11. HRESIMS of alcyopterosin U (**2**). Calculated for $C_{17}H_{21}NO_6H$, 336.1442.

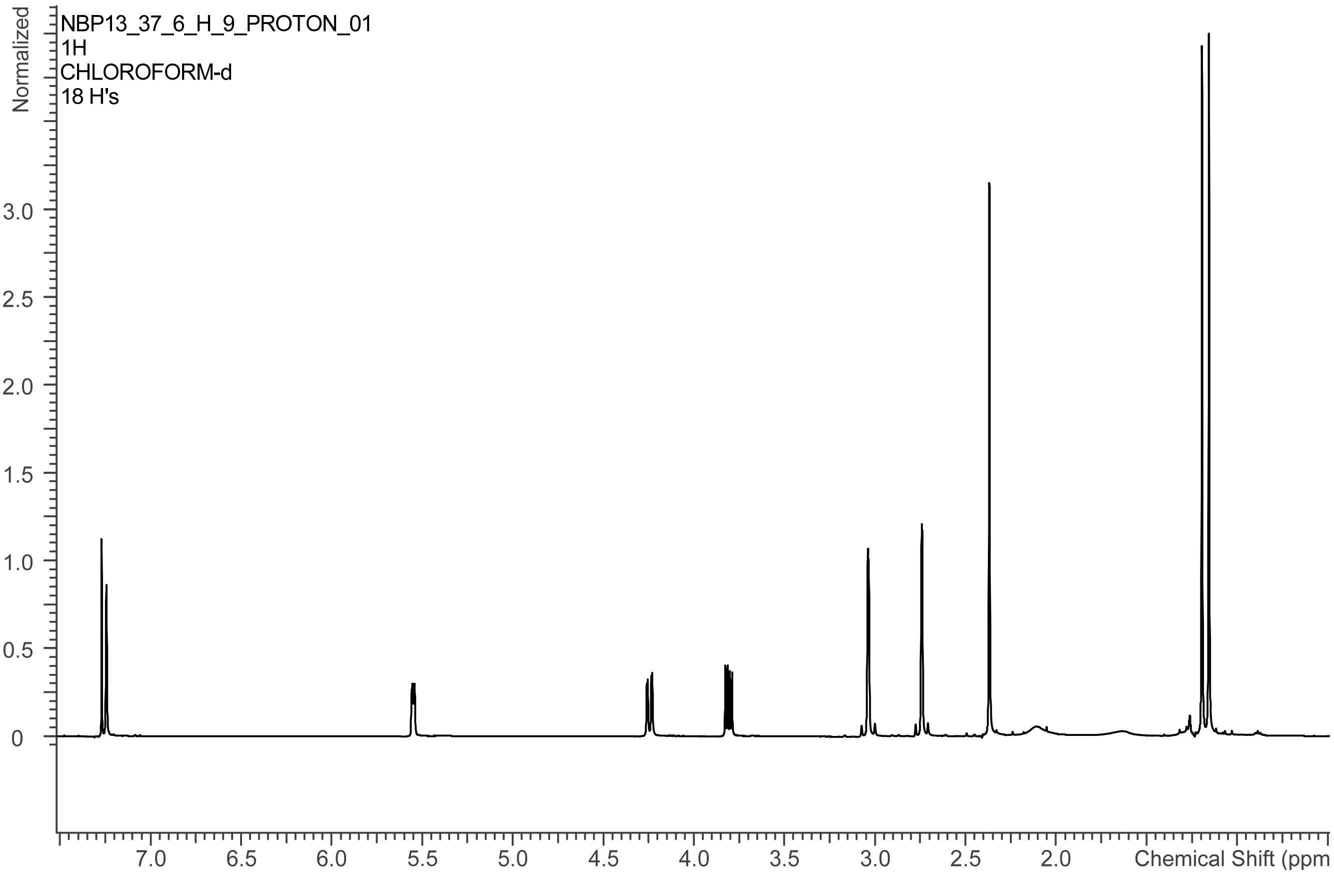

Figure S12. ^{1}H NMR spectrum of alcyopterosin V (**3**), 500 MHz, $CDCl_3$.

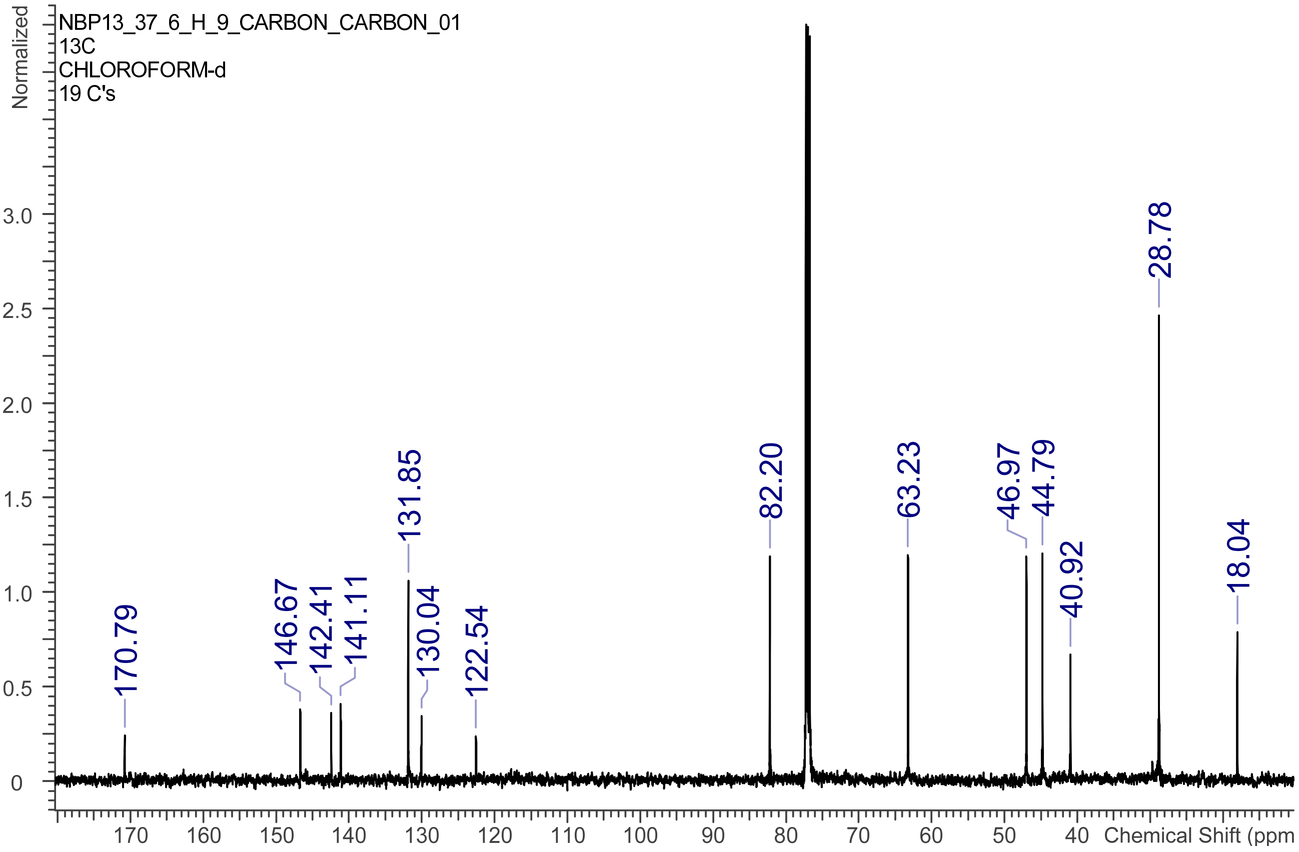

Figure S13. ^{13}C NMR spectrum of alcyopterosin V (**3**), 125 MHz, $CDCl_3$.

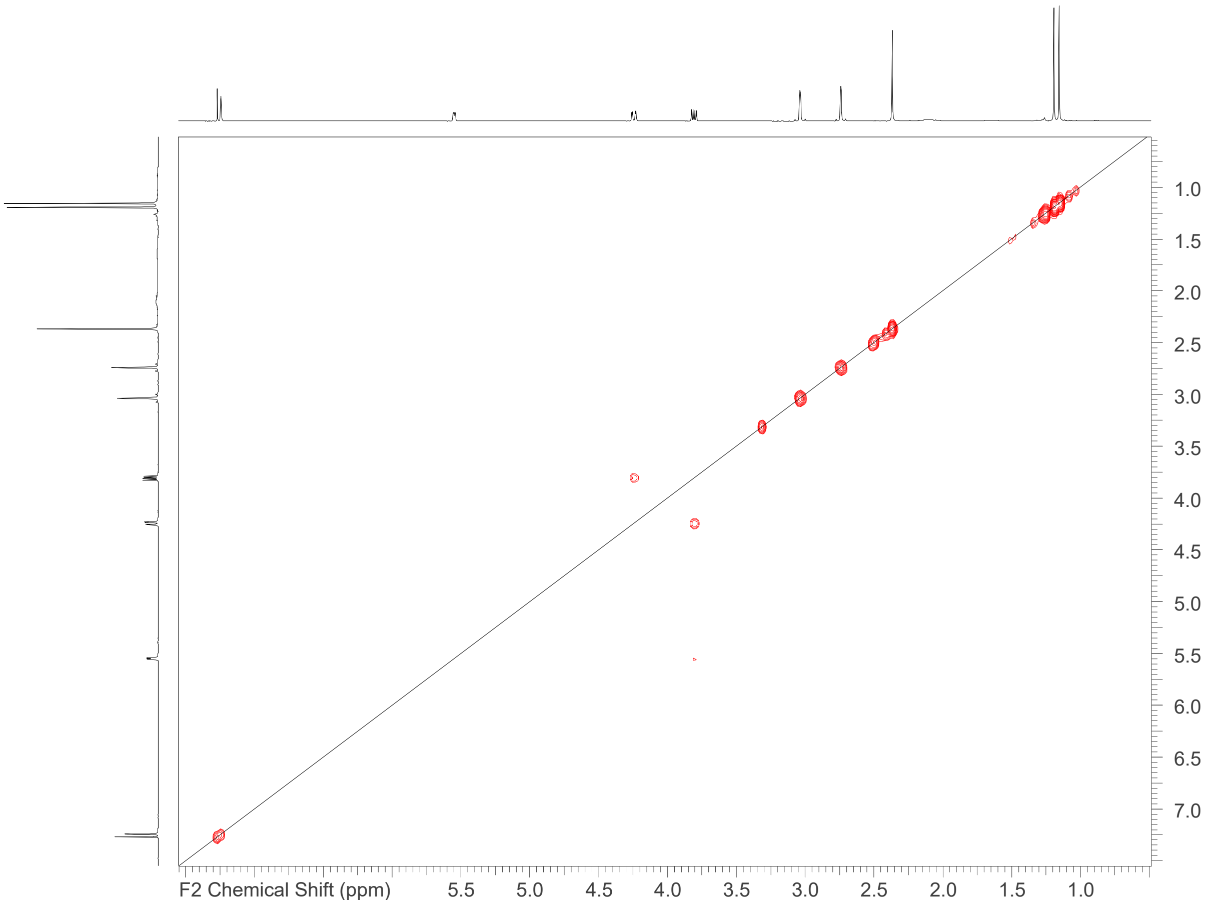

Figure S14. COSY spectrum of alcyopterosin V (**3**), 500 MHz, $CDCl_3$.

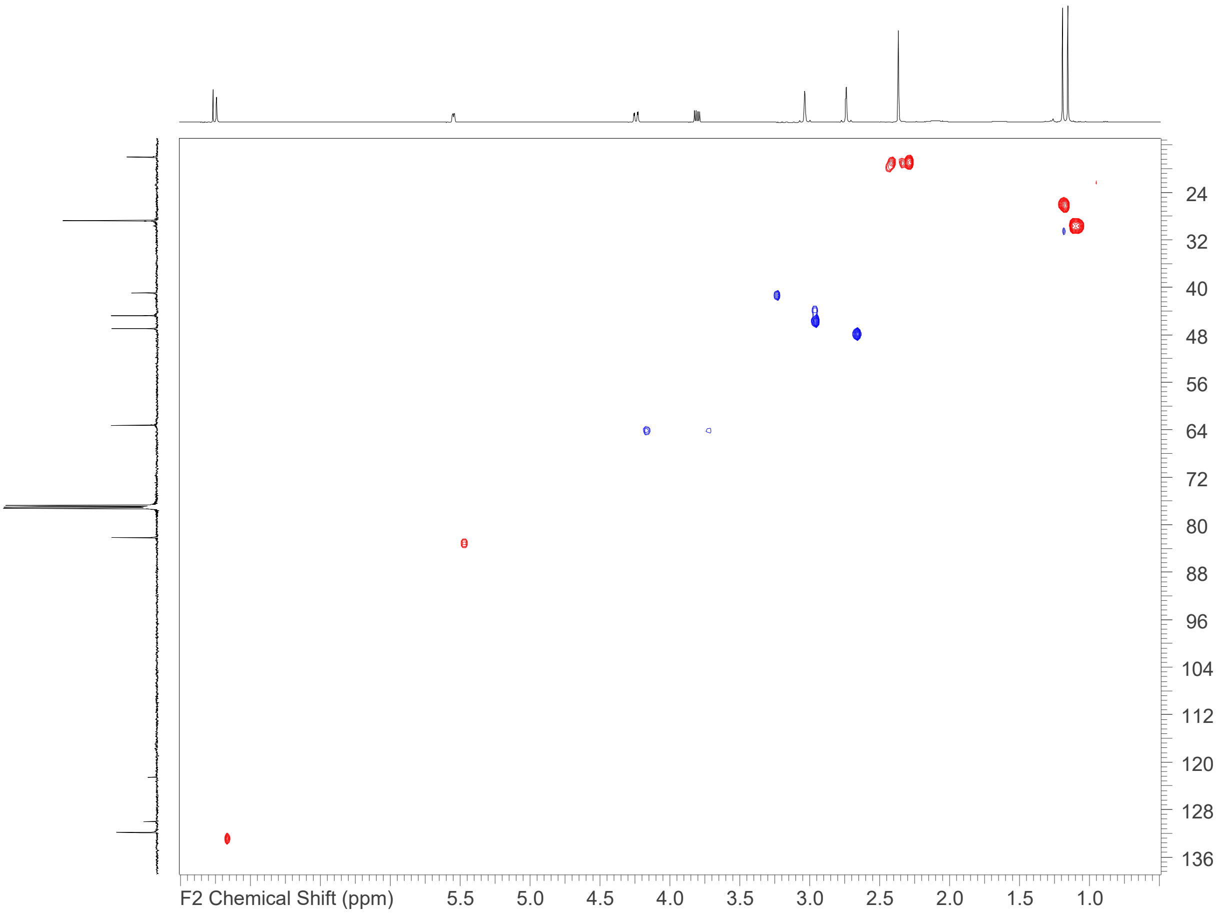

Figure S15. HSQC spectrum of alcyopterosin V (**3**), 500 MHz, $CDCl_3$.

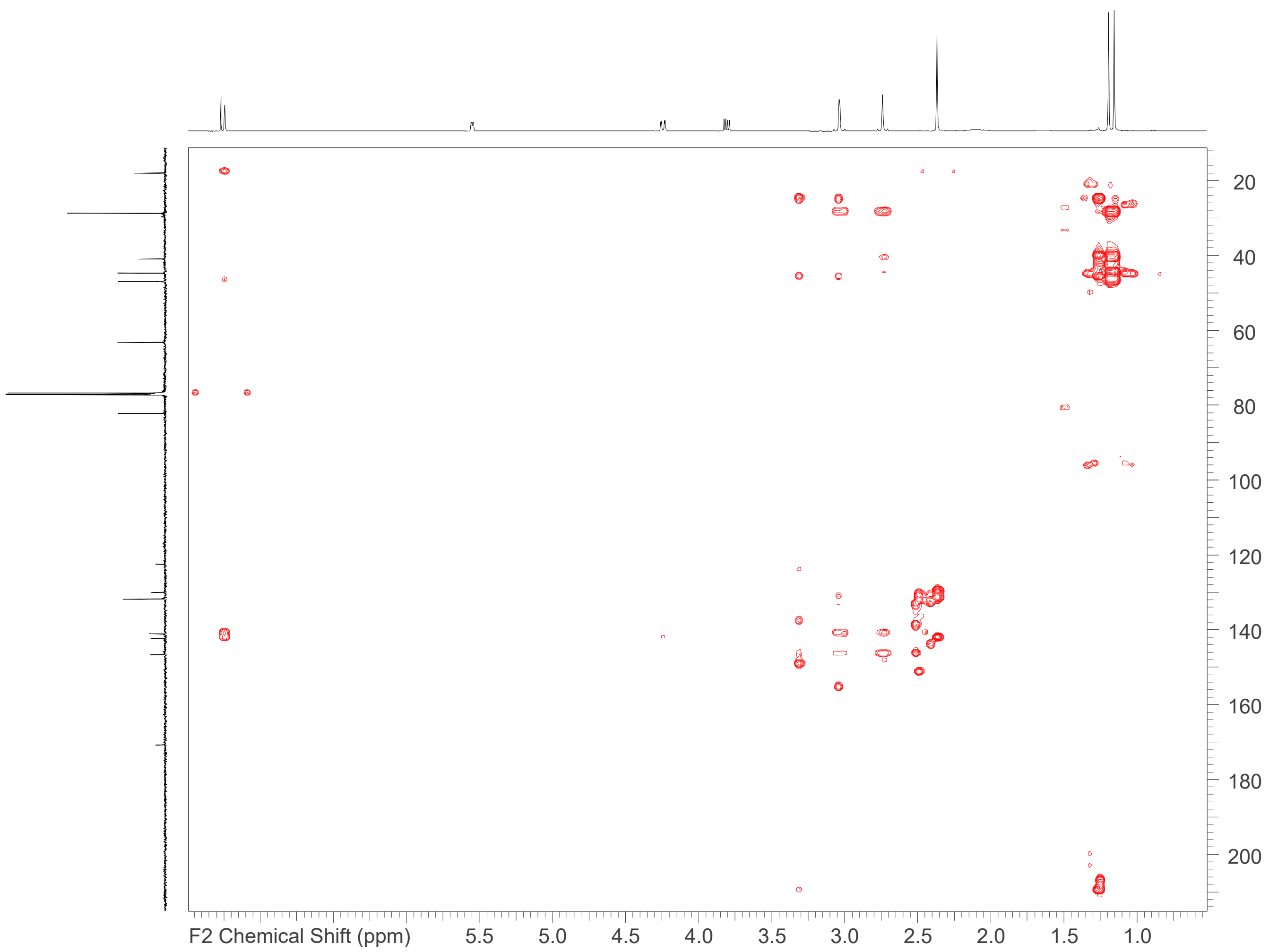

Figure S16. HMBC spectrum of alcyopterosin V (**3**), 500 MHz, $CDCl_3$.

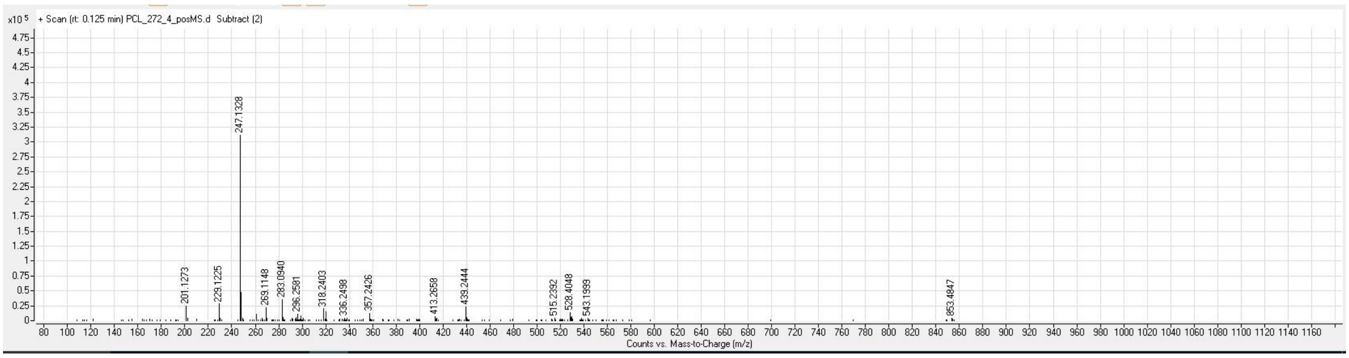

Figure S17. Blank subtracted HRESIMS of alcyopterosin V (**3**). Calculated for $C_{15}H_{18}O_3H$, 247.1329.

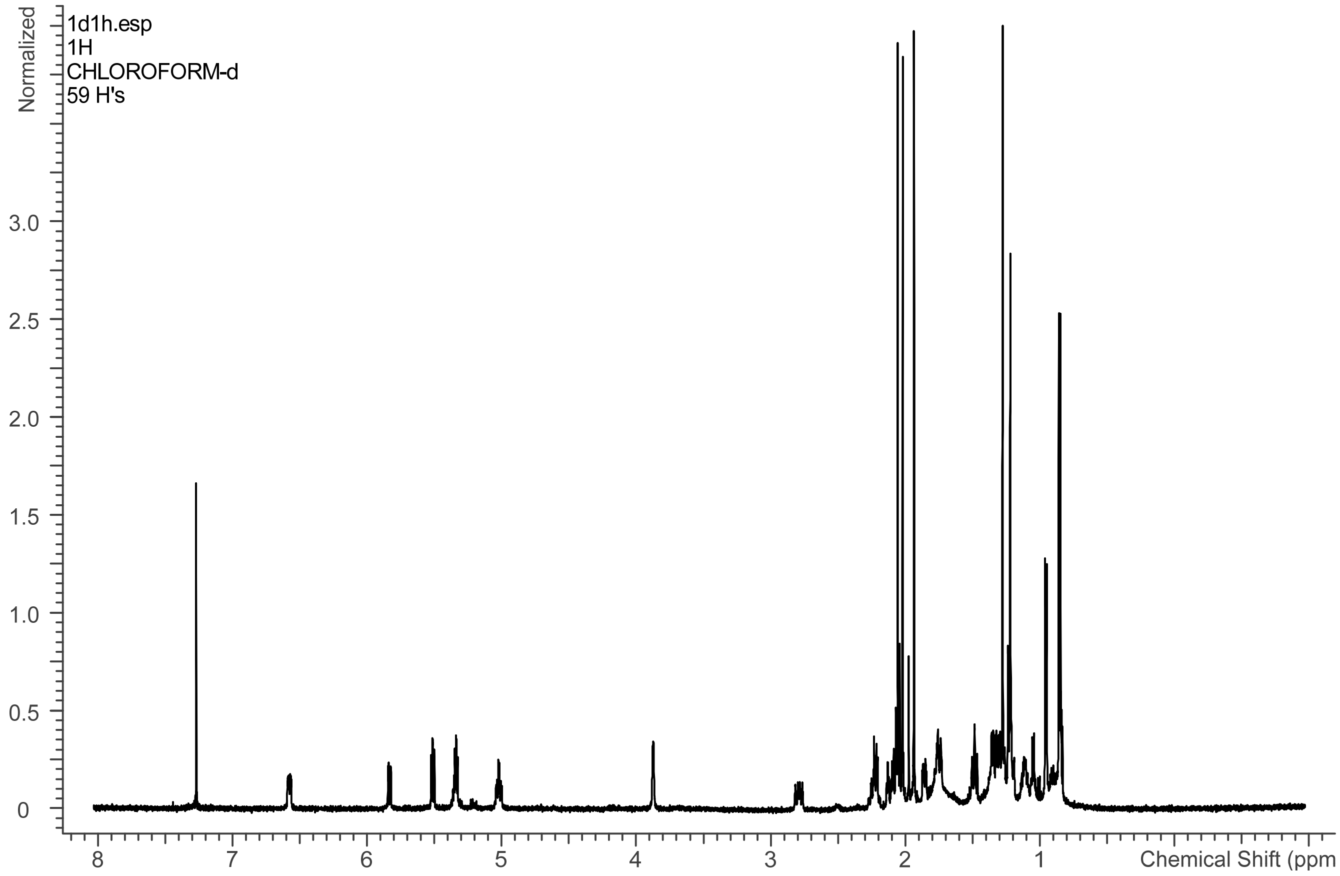

Figure S18. ^{1}H NMR spectrum of alcyosterone (**5**), 600 MHz, $CDCl_3$.

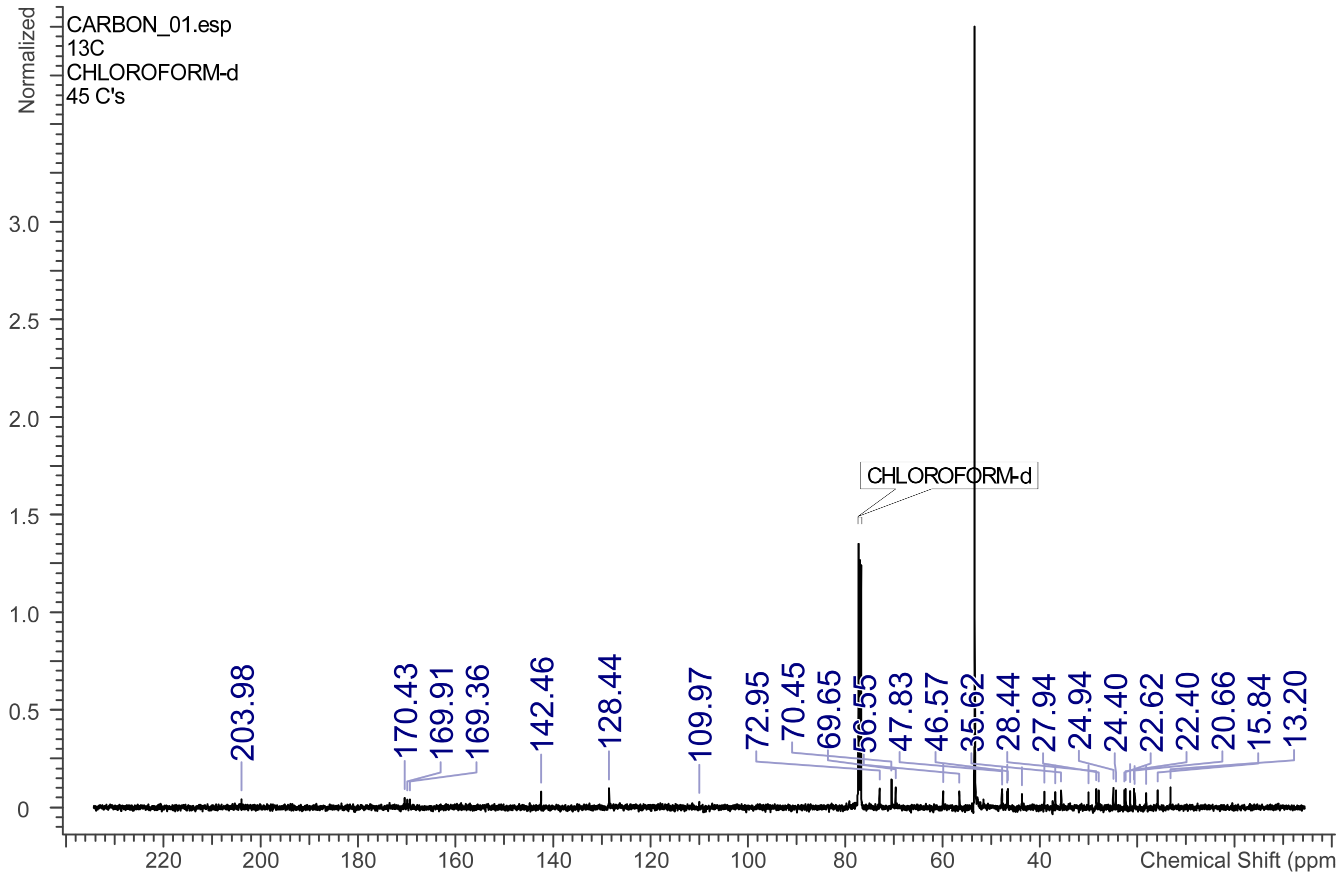

Figure S19. ^{13}C NMR spectrum of alcyosterone (**5**), 125 MHz, $CDCl_3$.

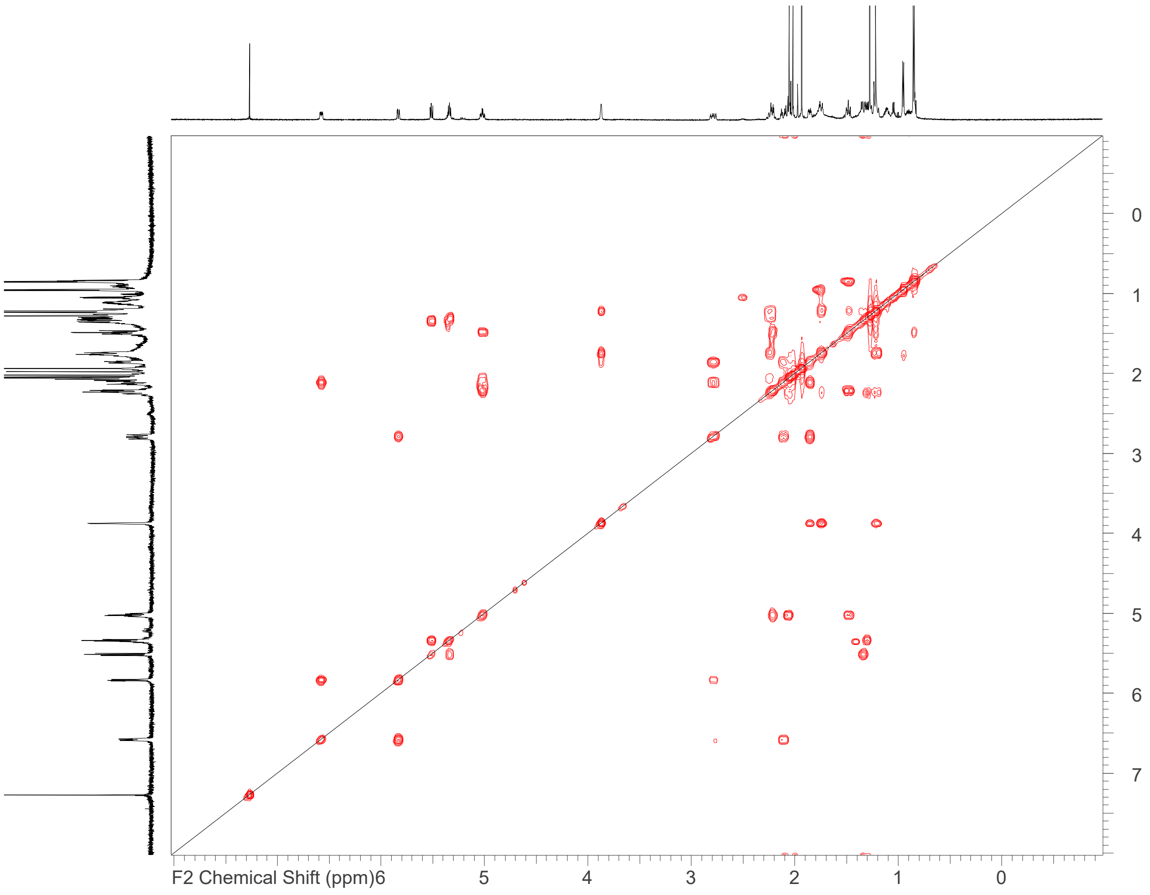

Figure S20. COSY spectrum of alcyosterone (**5**), 500 MHz, $CDCl_3$.

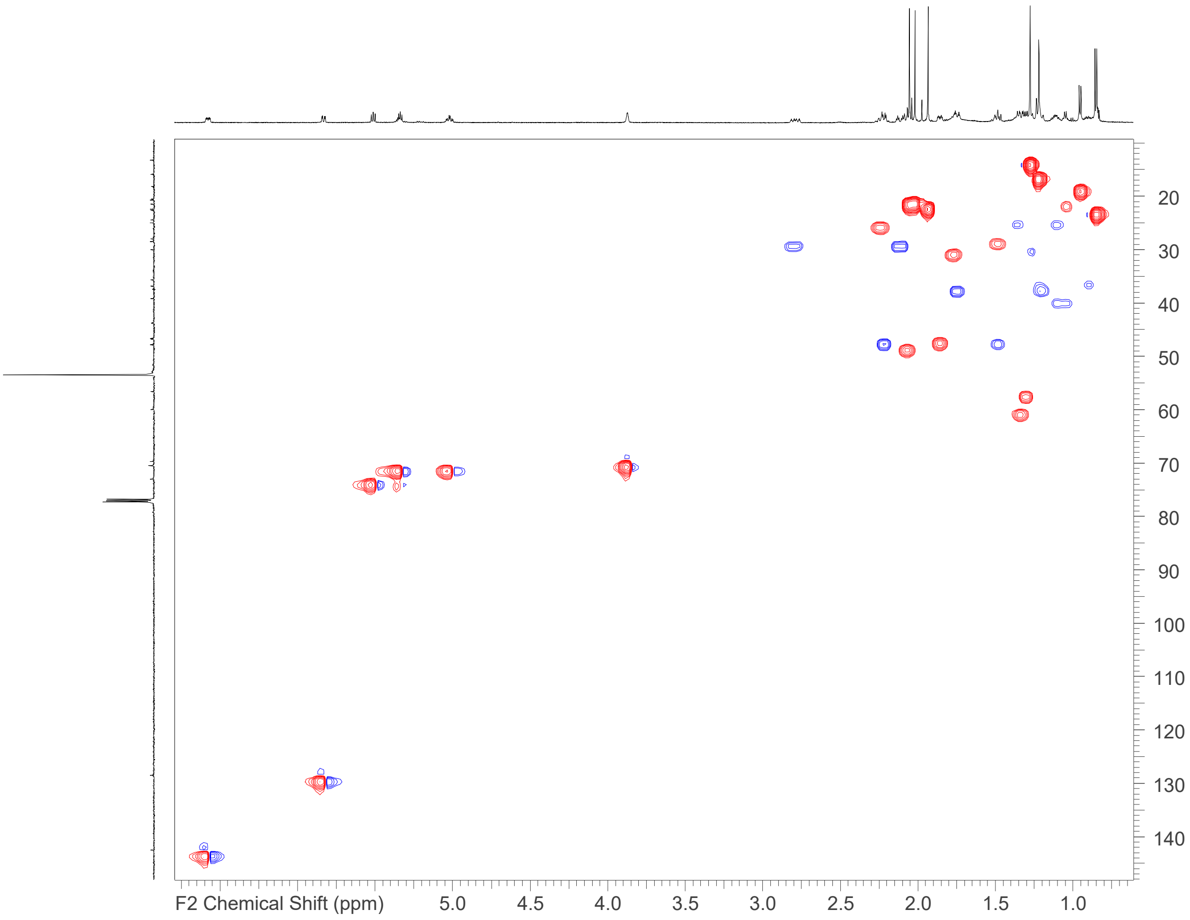

Figure S21. HSQC spectrum of alcyosterone (**5**), 500 MHz, $CDCl_3$.

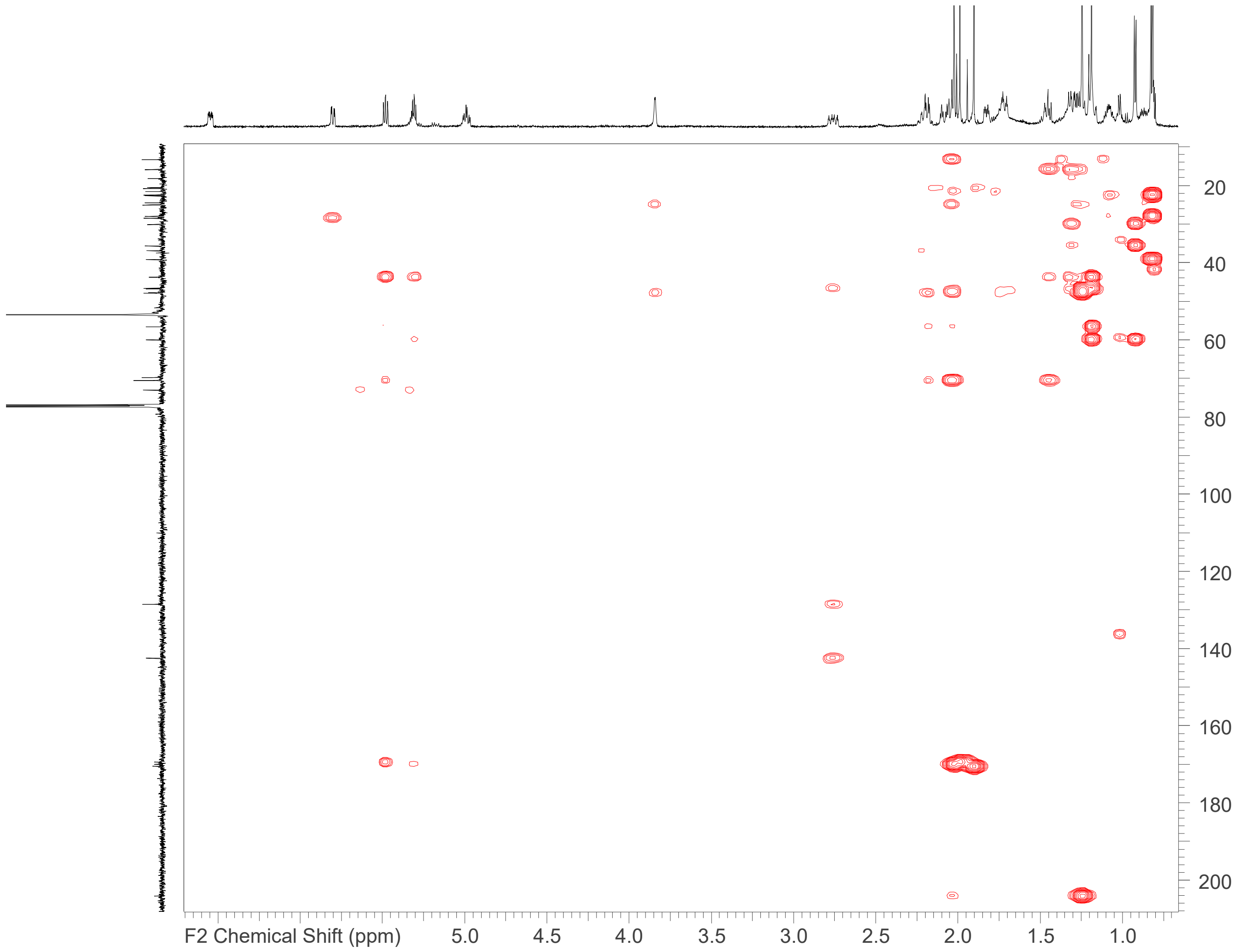

Figure S22. HMBC spectrum of alcyosterone (**5**), 500 MHz, $CDCl_3$.

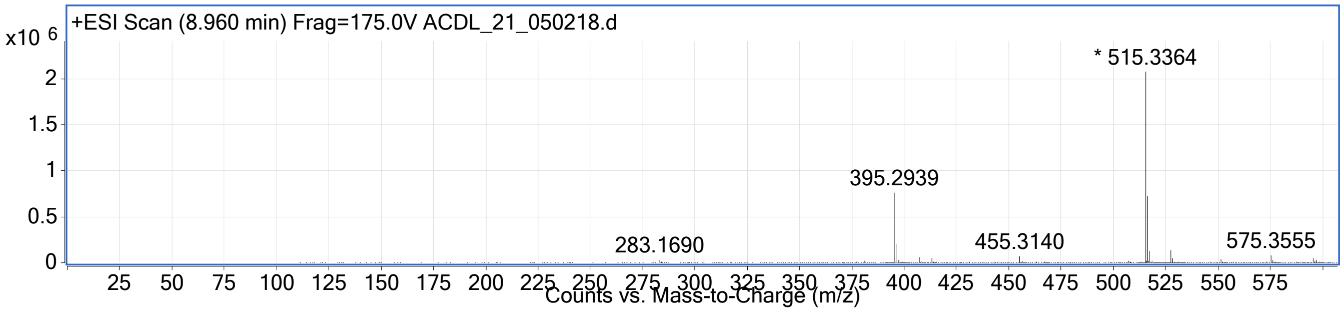

Figure S23. HRESIMS of alcyosterone (**5**). Calculated for $C_{33}H_{51}O_8$, *m/z* 575.3578 ($[M + H]^+$); calculated for $C_{31}H_{47}O_6$, *m/z* 515.3367 ($[M - OAc]^+$); calculated for $C_{27}H_{39}O_2$, m/z 395.2945 ($[M – OAc – 2HOAc]^+$).

Table S1. NMR shift comparison between compounds isolated in the current work to those previously published.

	Alcyopterosin E (**2**)				Alcyopterosin L				Alcyopterosin C				Alcyopterosin G				Bis(acetyl)alcyopterosin O			
	This work		Palermo [1]		This work		Palermo [1]		This work		Palermo [1]		This work		Palermo [1]		This work		Carbone [2]	
Position	δ_H	δ_C	δ_H	δ_C	δ_H	δ_C	δ_H	δ_C	δ_H	δ_C	δ_H	δ_C	δ_H	δ_C	δ_H	δ_C	δ_H	δ_C	$?_H$	$?_C$
1	3.03	44.7	3.04	45	2.92	44.0	2.92	44.0	2.87	42.3	2.87	42.3	2.79	46.3	2.79	46.3	2.72	46.4	2.74	46.4
					3.17		3.17													
2		148		148		147.7		150.8		149.4		149.7		141.5		141.5		142.4		142.4
3		122		122		123.2		123.1		133.9		134.6		135.0		135.0		135.5		135.5
4	4.57	76.6	4.58	77	3.88	42.1	3.88	42.1	4.53	70.2	4.53	70.6	4.60	72.6	4.60	72.6	4.14	63.9	4.15	63.9
	5.06		5.07		4.17		4.18													
5	5.66	71.6	5.67	72	5.70	79.6	5.70	79.5	3.19	27.7	3.18	27.8	3.17	26.8	3.17	26.8	3.03	29.7	3.03	29.7
6		140		141		139.8		139.8		138.7		139.4		130.6		130.6		132.8		132.8
7		130		130		130.8		130.8		136.2		136.7		135.5		135.5		130.3		130.3
8	7.27	132	7.27	132	7.47	131.8	7.48	131.8	7.46	122.9	7.46	123.5	7.00	127.2		127.2	7.01	127.7	7.01	127.7
9		142		142		144.2		144.2		133.9		134.0		143.0		143.0		142.4		142.4
10	2.75	47	2.75	47	4.71	82.8	4.71	82.8		211.0		211.4	2.70	47.7	2.70	47.7	2.69	47.6	2.69	47.6
11		41.1		41		45.3		45.3		45.8		45.5		39.7		39.7		39.7		39.7
12		170		170		169.6		169.6	2.32	15.0	2.32	25.4	4.69	60.4	4.69	60.4	5.15	62.1	5.15	62.1
13	2.40	17.9	2.41	18	2.40	18.0	2.40	18.0	2.42	20.4	2.42	20.2	2.34	19.9	2.34	19.9	2.35	20.0	2.35	20.0
14	1.19	28.7	1.19	29	1.19	26.7	1.20	26.7	1.23	25.2	1.23	25.4	1.15	29.0	1.15	29.0		29.0		29.0
15	1.15	28.7	1.16	29	1.07	21.5	1.08	21.4	1.23	25.2	1.23	25.4	1.15	29.0	1.15	29.0	1.14	29.0	1.14	29.0
4-*CH*$_3$CO																	2.06	21.0	2.06	21.0
4-CH$_3$*C*O																		170.6		170.6
12-*CH*$_3$CO																	2.07	21.0	2.07	21.0
12-CH$_3$*C*O																		170.9		170.9

[1]Palermo, J.A.; Brasco, M.F.R.; Spagnuolo, C.; Seldes, A.M. Illudalane sesquiterpenoids from the soft coral *Alcyonium paessleri*: The first natural nitrate esters. *J. Org. Chem.* **2000**, *65*, 4482-4486.

[2]Carbone, M.; Nunez-Pons, L.; Castelluccio, F.; Avila, C.; Gavagnin, M. Illudalane sesquiterpenoids of the alcyopterosin series from the Antarctic marine soft coral *Alcyonium grandis*. *J. Nat. Prod.* **2009**, *72*, 1357–1360

Table S2. Crystal data and structure refinement for alcyosterone (**5**).

Identification code	ACDL_21
Empirical formula	$C_{33}H_{50}O_8$
Formula weight	574.73
Temperature/K	100.0
Crystal system	orthorhombic
Space group	$P2_12_12_1$
a/Å	6.9203(5)
b/Å	14.3360(10)
c/Å	32.173(2)
α/°	90
β/°	90
γ/°	90

Volume/Å^3	3191.8(4)
Z	4
ρ_{calc}g/cm^3	1.196
μ/mm^{-1}	0.681
F(000)	1248.0
Crystal size/mm^3	0.042 × 0.036 × 0.018
Radiation	CuKα (λ = 1.54178)
2Θ range for data collection/°	5.494 to 137.154
Index ranges	-7 ≤ h ≤ 8, -16 ≤ k ≤ 17, -38 ≤ l ≤ 38
Reflections collected	32338
Independent reflections	5867 [R_{int} = 0.1453, R_{sigma} = 0.0776]
Data/restraints/parameters	5867/258/437
Goodness-of-fit on F^2	1.026
Final R indexes [I>=2σ (I)]	R_1 = 0.0659, wR_2 = 0.1389
Final R indexes [all data]	R_1 = 0.1009, wR_2 = 0.1577
Largest diff. peak/hole / e Å^{-3}	0.40/-0.26
Flack parameter	-0.10(19)

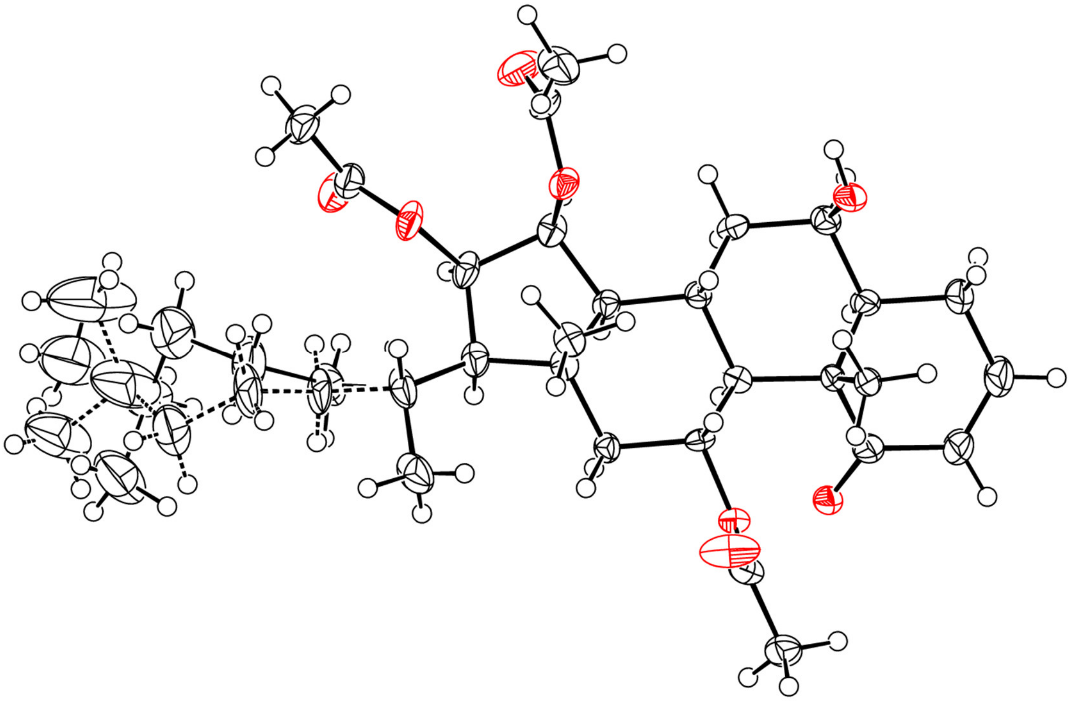

Figure S24. Asymmetric unit of alcyosterone (**5**). Anisotropic displacement parameters drawn at 50% probability level.
